# Differential effects of Foxp2 disruption in distinct motor circuits

**DOI:** 10.1038/s41380-018-0199-x

**Published:** 2018-08-14

**Authors:** Catherine A. French, María F. Vinueza Veloz, Kuikui Zhou, Saša Peter, Simon E. Fisher, Rui M. Costa, Chris I. De Zeeuw

**Affiliations:** 1 Champalimaud Research, Champalimaud Centre for the Unknown, Lisbon, Portugal; 2 Department of Neuroscience, Erasmus MC, Rotterdam, The Netherlands; 3 School of Medicine, Escuela Superior Politécnica de Chimborazo, Riobamba, Ecuador; 4 The Brain Cognition and Brain Disease Institute, Shenzhen Institutes of Advanced Technology, Chinese Academy of Sciences, Shenzhen, Guangdong, China; 5 Language and Genetics Department, Max Planck Institute for Psycholinguistics, Nijmegen, The Netherlands; 6 Donders Institute for Brain, Cognition and Behaviour, Radboud University, Nijmegen, The Netherlands; 7 Department of Neuroscience, Zuckerman Mind Brain Behavior Institute, Columbia University, New York, USA; 8 Netherlands Institute for Neuroscience, Royal Netherlands Academy of Arts and Sciences (KNAW), Amsterdam, The Netherlands

**Keywords:** Neuroscience, Genetics

## Abstract

Disruptions of the *FOXP2* gene cause a speech and language disorder involving difficulties in sequencing orofacial movements. FOXP2 is expressed in cortico-striatal and cortico-cerebellar circuits important for fine motor skills, and affected individuals show abnormalities in these brain regions. We selectively disrupted *Foxp2* in the cerebellar Purkinje cells, striatum or cortex of mice and assessed the effects on skilled motor behaviour using an operant lever-pressing task. Foxp2 loss in each region impacted behaviour differently, with striatal and Purkinje cell disruptions affecting the variability and the speed of lever-press sequences, respectively. Mice lacking Foxp2 in Purkinje cells showed a prominent phenotype involving slowed lever pressing as well as deficits in skilled locomotion. In vivo recordings from Purkinje cells uncovered an increased simple spike firing rate and decreased modulation of firing during limb movements. This was caused by increased intrinsic excitability rather than changes in excitatory or inhibitory inputs. Our findings show that Foxp2 can modulate different aspects of motor behaviour in distinct brain regions, and uncover an unknown role for Foxp2 in the modulation of Purkinje cell activity that severely impacts skilled movements.

## Introduction

The discovery that disruptions of one copy of the *FOXP2* gene cause a severe speech and language disorder has generated substantial interest in elucidating the neural functions of the encoded protein. Initial findings came from a large multigenerational family (the KE family), where a missense mutation was found to be responsible for deficits in many facets of speech and language [1–3]. A number of other familial and de novo cases of *FOXP2*-related disorders have since broadened the genotypic and clinical spectrum [4–6]. Affected individuals display wide^−^ranging impairments in oral and written language, which impact on both receptive and expressive skills. Less severe deficits are evident in aspects of non-verbal cognition [3, 6]. A core phenotype of the disorder is developmental verbal dyspraxia (also known as childhood apraxia of speech), where imprecise and inconsistent neural control of sequences of orofacial movements impedes development of fluent speech [2, 3, 6]. Gross motor functions outside of the orofacial region, such as limb praxis, appear relatively spared [3, 6]. However, fine motor-skill deficits have been reported in some individuals with FOXP2 disruptions [5, 7], and affected KE family members show reduced performance when tapping out rhythms [8].

FOXP2 is highly conserved in many vertebrates and functions as a transcription factor, modulating expression of target gene networks that regulate processes such as neural development, synaptic plasticity and neurite outgrowth [9–12]. Its expression pattern in the brain is broadly concordant across species and shows some intriguing overlaps with circuits that have known motor-related functions. Most FoxP2 functional studies have been carried out in humans, rodents or songbirds where the protein is found in neuronal subpopulations of the cerebral cortex, basal ganglia, and thalamus, as well as in the deep nuclei and Purkinje cells of the cerebellum and the inferior olive of the medulla oblongata [13–15] (here we use the standard nomenclature FOXP2 for humans, Foxp2 for mice and FoxP2 for other species, or when referring to several species; genes and RNA are italicised). Structural neuroimaging of the KE family uncovered subtle but significant alterations in grey-matter density in affected members in several of these same areas, including the caudate nucleus, ventral cerebellum and inferior frontal gyri [16]. Moreover, functional imaging during language-based tasks showed reduced activation in brain regions that include Broca’s area (left inferior frontal gyrus) and the putamen [17].

Valuable insights have come from Foxp2 mouse mutants and knockouts [18, 19], as well as FoxP2 manipulations in songbirds [18, 20]. Mice that are homozygous for the KE-family mutation or for knockout alleles show severe developmental delay, motor dysfunction and postnatal lethality, dying 3–4 weeks after birth [21–23]. They also have a disproportionately small cerebellum, with decreased foliation, although cellular organisation is broadly intact [21, 22]. In contrast, mice with heterozygous disruptions develop normally and have no obvious neural pathology. However, they display motor-skill learning deficits on the accelerating rotarod [22, 24] and on a tilted running-wheel system [22]. Adult males, heterozygous for the KE-family mutation (*Foxp2-R552H/*+), also show altered sequencing of ultrasonic vocalisation (USVs), which in mice are typically innately produced [25]. Electrophysiology studies of *Foxp2-R552H/*+ animals uncovered strongly impaired long-term depression at glutamatergic striatal inputs [22] and abnormally high striatal activity, which is aberrantly modulated as mice learn to run on the rotarod [24]. In zebra finches, FoxP2 knockdown in the striatal song nucleus area X disrupts developmental and social modulation of song variability [26, 27]. Foxp2 function in the cerebellum has been less well studied. Subtle alterations in cerebellar plasticity at parallel fibre to Purkinje cell synapses were found in *Foxp2-R552H/*+ mice [22]. Recently, Foxp2 knockdown in the embryonic cerebellum was shown to lead to altered motor function (righting reflex and negative geotaxis) and reduced USVs in neonatal animals [28].

Learning to perform organised sequences of movements is critical for a multitude of complex behaviours including speech. Initiation and termination of motor-sequences is compromised in disorders such as Parkinson’s and Huntington’s disease [29], and the coordination of these sequences is affected in cerebellar ataxia [30]. The cerebellum, basal ganglia and cortex are the major brain regions implicated in the learning and performance of motor-sequences, and make complementary contributions to motor behaviour [31]. The cerebellum functions in error correction and control of ongoing movement, whilst the striatum is important for reinforcement learning and motor “chunking” (the process by which sequences of motor actions are concatenated with training [29]). The motor cortex stores representations of learned sequences and is involved in action planning [31]. As noted above, FoxP2 is enriched in particular neuronal subpopulations of these brain regions [13–15], and affected KE-family members also show structural and functional abnormalities in these areas [16, 17]. It is highly likely that Foxp2 contributes differently to motor behaviour depending on its expression site, but to date it has not been possible to identify region-specific or cell-type specific functions of the protein.

Here we used a conditional approach to make homozygous Foxp2 disruptions in the three brain regions of interest and assessed the effects on skilled motor behaviour. This is a strategy, which has been successfully used by others to dissect out functions of broadly-expressed proteins implicated in neurodevelopmental disorders [32, 33]. Loss of Foxp2 in these defined regions impacted motor-skill learning and performance differently, with Foxp2 disruption in the cerebellum and striatum affecting the speed and variability of lever-press sequences, respectively. Mice lacking Foxp2 in Purkinje cells executed lever-press sequences more slowly and also showed deficits in skilled walking on the ErasmusLadder, a system for testing locomotor learning [30]. Moreover, in vivo recordings from Purkinje cells in these animals during rest and locomotion uncovered changes in simple spike firing rate and modulation. Whole-cell recordings revealed that synaptic excitation and inhibition are normal, but that increased intrinsic excitability of Purkinje cells, resulting from Foxp2 protein loss in these neurons, is the plausible mechanism for the aberrant simple spike modulation observed in vivo.

## Materials and methods

### Animals

Work in Portugal was approved by the Direcção Geral de Veterinária and experiments in The Netherlands were approved by the National DEC (Dier-Experimentele-Commissie) at Erasmus MC. The Foxp2 conditional line is available from The Jackson Laboratory Repository (stock No. 026259), and was generated and maintained on a C57BL/6J genomic background [21]. All Cre lines were backcrossed to C57BL/6J, but the areas flanking the genetically modified regions may still have harboured DNA originating from other genomic backgrounds. *Foxp2-flox/flox* mice were crossed to *L7-Cre* [34], *Rgs9-Cre* [35] or *Emx1-Cre* [36] mice to achieve Foxp2 deletion in cerebellar Purkinje cells, striatal medium-sized spiny neurons or the cerebral cortex, respectively. *Foxp2-flox/flox Cre* positive animals were always compared to *Foxp2-flox/flox* littermate controls. *Cre* expression begins postnatally in the *L7-Cre* and *Rgs9-Cre* lines and embryonically in the *Emx1-Cre* line. In mice which express *LacZ* under control of the *L7* promoter, reporter gene expression is seen in all Purkinje cells by P9 and in four parasagittal stripes at earlier time points [37]. When *Emx1-Cre* and *Rgs9-Cre* mice are crossed with a Cre-dependent reporter strain (R26R), robust recombination levels are observed at E10.5 and P8, respectively [35, 36]. Mice used for the operant motor-sequence learning, ErasmusLadder and accelerating rotarod tasks as well as for immunohistochemistry and in vitro electrophysiology were 10 weeks to 6 months old. Mice used for in vivo extracellular recordings were 15 weeks to 1 year old. Male and female animals were used for all behavioural experiments, whereas only males were used for electrophysiological recordings. Previous whole-cell recordings of Purkinje cells in C57BL/6J mice did not reveal any difference in the parallel fibre to Purkinje cell input between males and females [38]. Sample size was chosen based on studies using related methods and is similar to what is generally employed in the field. Randomisation was not used to assign animals to experimental groups, and the investigator was not blinded to the genotype of animals while running experiments.

### Immunohistochemistry and histology

Mice of between 12 weeks and 6 months of age were deeply anaesthetised and transcardially perfused with 4% paraformaldehyde (PFA). Brains were removed and post-fixed for 2 h in 4% PFA at room temperature before being transferred to 10% sucrose overnight. Samples were then embedded in gelatin blocks (11% gelatin/10% sucrose), post-fixed for 2 h in 10% formaldehyde/ 30% sucrose and placed overnight in 30% sucrose. Next, 40 µm sections were cut on a freezing microtome. The sections were either directly stained with Cresyl Violet for quantification purposes and/ or used for Foxp2 immunohistochemistry, which was carried out with the Vectastain Elite ABC peroxidase kit (Vector Laboratories, Burlingame, CA, USA). Sections were incubated with Foxp2 antibody (1:1500, sc−21069, goat N16, Santa Cruz Biotechnology Inc., Santa Cruz, CA, USA), and immunostaining was visualised using diaminobenzidine. Immunoperoxidase-stained sections were analysed and photographed using a Leica DM-RB microscope and DC300 digital camera. The morphology of granule cells was examined using Golgi staining. Dendrite numbers were counted for five cells per animal blind to genotype. A Neurolucida system linked to an Olympus microscope (operating at a magnification of 10 × 2.5) was used to count Cresyl Violet stained cell bodies and/ or Foxp2-expressing cells as well as to measure the length of the Purkinje cell layer in cerebellar lobules III, VI and IX and surface areas of the striatum and cerebral cortex (using 3–4 representative sections per animal). Cell counting was performed with the investigator blinded to genotype.

### Operant motor-sequence learning task

Mice of between 10 weeks and 6 months of age were trained in operant chambers housed in sound attenuating boxes (Med-Associates, St. Albans, VT) as previously described [39]. A lever was extended 4.5 cm to the left of a food magazine containing a metal cup into which 20% sucrose solution was delivered from a syringe pump. Lever presses, syringe pump activations, head entries into the food magazine (detected by infrared beam) and licks in the cup (detected by contact lickometer) were recorded as timestamps with a 10 ms resolution. Two days before training began, mice were placed on food restriction, which continued throughout training and maintained animals at around 85% of their original body weight. Overnight water restriction was also imposed. Mice received one daily training session that was scheduled as follows:

Habituation (1 day): mice were placed in the operant chambers for 90 min. Magazine approach training (1 day): 30 sucrose reinforcers were delivered to the food magazine on a random time schedule (average 1 reinforcer/ min). Continuous reinforcement (CRF) (4 days): the lever was extended and a reinforcer was delivered after each press. The possible number of reinforcers earned increased across training days (CRF5, 15, 30, 30), and a session was completed after the specified number of reinforcers had been delivered or 90 min elapsed. Most mice learned to associate lever pressing with reinforcer delivery within 4 days, a few animals required additional CRF30 training sessions, but all were eventually able to perform the task. Self-paced fixed-ratio 8 (FR8) (12 days): a reinforcer was delivered after eight presses and mice could earn up to a total of 30 per session. High-speed FR8 (maximum of 9 days): a reinforcer was delivered after eight presses were completed within a specified time, which became progressively faster as training progressed (first day: eight presses in 16 s; second day: eight presses in 12 s; subsequent days: eight presses in 8, 6, 4, 2, 1, 0.5 and 0.25 s). Animals’ training ceased when they failed to earn any reinforcers in a session.

Data analyses of the operant motor-sequence learning task were performed using custom-written Matlab code (Mathworks, Natick, MA, USA) (code available on request). In the self-paced training phase sequences of lever presses were broken by either a bout of ≥10 licks (assumed to be sucrose consumption) or a time period of ≥20 s (mouse assumed to have become disengaged from the task). The latter value was chosen based on the statistics of lever pressing for each animal as previously described [39]. In the high-speed training phase the time period used to break press sequences was adjusted to ≥6 s. The following features were then calculated for each sequence—length (number of presses in a sequence), duration (time between the first and last presses of a sequence), inter-sequence interval (time between the last press of one sequence and the first press of the next) and within-sequence inter-press interval (IPI) (average time between adjacent presses in a sequence). IPIs were divided into three groups as follows: rapid IPIs (no event between presses), check IPIs (presses separated by head entry into the food magazine and ≤10 licks in the sucrose cup), and consumption IPIs (presses separated by head entry and >10 licks). Ultrafast IPIs were defined as being ≤0.25 s. The median and median absolute deviation (MAD)/median (a measure of variability) were calculated for each IPI group. These non-parametric measures were chosen because IPI distributions were frequently heavy tailed (possibly reflecting when animals become disengaged from the task), which can strongly influence metrics such as the mean.

Statistical analyses were conducted in SPSS (SPSS Inc., Armonk, NY, USA). Kolmogorov-Smirnov and Shapiro-Wilk tests were used to determine if data were normally distributed and Levene’s test was used to assess variation between groups. Where necessary data were transformed. All data were subsequently analysed by repeated measures ANOVA and if the assumption of sphericity was violated (assessed using Mauchly’s test) a Greenhouse-Geisser correction was applied. When genotype effects were statistically significant (*α* = 0.05) Fisher’s LSD post hoc tests were performed. In the high-speed training phase, mice were removed from the experiment when they did not receive a reinforcer during a session. In order to use repeated measures ANOVA, missing values were replaced by the mean of the remaining animals. This was done until FR8 2 s after which many animals dropped out and it was not possible to perform meaningful statistics. The numbers of animals remaining at each speed of the high-speed training phase are summarised in Supplementary Table 1.

### ErasmusLadder

Details of the fully automated ErasmusLadder and associated software can be found in references [40, 41]. The ErasmusLadder consists of a horizontal ladder between two shelter boxes, each equipped with an LED spotlight in the roof and two pressurised air outlets in the back. Sensory stimuli (light and air) serve to control the moment of departure of the mice. The ladder itself has 37 rungs on each side, and each rung can be displaced vertically following a command from the control system. Even-numbered rungs on one side and odd-numbered rungs on the other were elevated by 6 mm, thereby creating a left/right alternating pattern. All rungs are equipped with custom-made pressure sensors that are continuously monitored. The setup is controlled by software written in LabView (National Instruments, Austin, TX, USA) that operates with a fixed cycle of 2 ms. For the current study, we followed a paradigm similar to that of a previous study described in reference [41]. Each mouse had to perform one daily session for 8 days, with 2 days of rest in the middle (i.e. between sessions 4 and 5). Each daily session consisted of 72 trials during which the mouse had to walk back and forth between the two shelter boxes. During the first four sessions (“unperturbed sessions”) we assessed naive locomotion where none of the rungs moved. During the last four sessions (i.e. sessions 5–8), we tested locomotion adaptation by challenging the mouse to deal with the appearance of an obstacle, which was preceded by a tone 200 ms prior to its occurrence (“perturbed sessions”). The obstacle was induced by elevating one of the lower rungs by 18 mm, thus creating an obstacle of 12 mm just in front of the mouse. The location of the obstacle on the ladder varied randomly between trials, but it always appeared on the animal’s right side (regardless of walking direction). The exact timing of the obstacle appearance depended on the walking pattern and the predicted trajectory of the mouse (for details see reference [40]). Steps were recorded as touches on the rungs; to prevent false positives, we took into account only touches that lasted >30 ms. To avoid detecting hind limb touches as backward steps, we accepted only sequences of two or more consecutive backward steps as true backward movements. The analyses of forward steps revealed that mice usually step from one elevated rung to the next, skipping the lower rung (i.e. step length = 2), or to the consecutive elevated rung, skipping three rungs (i.e. step length = 4). Hence, we considered steps with a step length equal to 2 or 4 to be “small regular steps” or “large regular steps”, respectively. Other step lengths, including missteps (i.e. stepping from or to a lower rung), leaps (i.e. step lengths > 4) as well as backward steps, occurred less frequently and were therefore termed “irregular steps”. To reduce the potential impact of a putative bias due to the air and / or light stimuli in the shelter box, the first and last step of each trial (i.e. stepping out of and into the shelter boxes) were omitted from analyses.

Data collected from the ErasmusLadder were stored in a relational database (MySQL, Oracle, Redwood Shores, CA, USA) and then processed off-line using custom-written software in LabView and Python (code available on request) (Python Software Foundation, Beaverton, OR, USA). Step lengths were determined by the distance between two consecutive touches. Likewise, step time was defined as the time that elapsed between the onsets of two consecutive touches of the same limb. To estimate skilled locomotion we calculated the percentage of trials per session in which there were at least two or more missteps (i.e. stepping from or to a lower rung more than once). Statistical analyses were conducted in SPSS (SPSS Inc., Armonk, NY, USA). All data were subsequently analysed with repeated measures ANOVA. Data sets were analysed with the investigator blinded to the genotype of the animals.

### Accelerating rotarod

Animals of less than 32 g were used for this task, where body weight and latency to fall are not correlated [42]. A stand-alone rotarod (ENV-577M, Med-Associates, St Albans, VT, USA) was set to accelerate from 6 to 60 r.p.m. over a 300 s time period. Mice were trained for five consecutive days, with one daily session consisting of ten trials separated by 300 s intervals. Mice were placed on the rotarod and trials were deemed to have started when the rod began to turn. Trials ended when mice fell from the rod or after 300 s elapsed.

### In vitro electrophysiology

After the decapitation of mice under isoflurane anaesthesia, the cerebellum was put in a cold slicing medium containing (in mM) 240 sucrose, 2.5 KCL, 1.25 Na_2_HPO_4_, 2 MgSO_4_, 1 CaCl_2_, 26 NaHCO_3_ and 10 D-Glucose, this solution was carbogenated continuously (95% O_2_ and 5% CO_2_). Sagittal slices, 250 μm thick, of the cerebellar vermis were cut using a vibrotome (VT1200S, Leica) and put in carbogenated artificial cerebrospinal fluid (ACSF) containing (in mM): 124 NaCl, 5 KCL, 1.25 Na_2_HPO_4_, 2 MgSO_4_, 2 CaCl_2_, 26 NaHCO_3_ and 20 D-Glucose, for approximately 1 h (34 ± 1 °C) before the start of the experiment. Slice physiology was done at room temperature 21 ± 1 °C or 33 ± 1 °C as indicated in the results section and in the presence of 100 μM picrotoxin except for sIPSCs recordings. Whole-cell patch clamp recordings were performed with an EPC9 amplifier (HEKA Electronics, Lambrecht, Germany). Action potential threshold (identified by steepest slope in membrane potential prior to action potential) and AHP amplitude (minimal membrane potential relative to the action potential threshold) was calculated using Clampfit software (Molecular Devices). The whole-cell recordings of Purkinje cells (PCs) were visualised with an upright microscope (Axioskop 2 FS, Carl Zeiss) equipped with a 40X objective. Recording electrodes (3–5 MΩ, 1.65 mm OD and 1.11 mm ID, World Precision Instruments, Sarasota, FL, USA) were filled with an intracellular solution containing (mM): 120 K-Gluconate, 9 KCL, 10 KOH, 4 NaCL, 10 HEPES, 28.5 Sucrose, 4 Na_2_ATP, 0.4 Na_3_GTP (pH 7.25–7.35 with an osmolarity of 295 ± 5). For the recording of spontaneously occurring inhibitory postsynaptic currents (sIPSCs) we used an intracellular solution containing (mM): 150 CsCl, 1.5 MgCl_2_, 0.5 EGTA, 4 Na_2_ATP, 0.4 Na_3_GTP, 10 HEPES, 5 QX314 (pH 7.25–7.35 with an osmolarity of 295 ± 5). For the extracellular stimulation of parallel fibres (PFs), similar patch electrodes were filled with ACSF and positioned in the upper third of the molecular layer lateral to the patched Purkinje cell.

For the evaluation of PF to PC transmission (21 ± 1 °C) we used various stimulation intensities (3–12 µA, with 3 µA increments) and inter-stimulus intervals (50–200 ms, 50 ms increments). For recordings of sIPSCs we used the previously mentioned Cs-based internal solution and recorded (33 ± 1 °C) their occurrence during at least 60 s. The intrinsic excitability protocol (33 ± 1 °C) was performed using different current injections (100–1000 pA, 100 pA increments) at the level of the PC soma while the spike count was quantified as a measure of excitability. Data sets were analysed with the investigator blinded to the genotype of the animals.

### In vivo extracellular recordings

Male mice of between 15 and 30 weeks old were prepared for recordings as described previously [43]. In short, under general anaesthesia, a pedestal was fixed on the skull overlying the frontal and parietal bones of the animal, and a recording chamber was placed around a small craniotomy in the left occipital bone. After 2 days of recovery, animals were habituated on a foam wheel for about half an hour every day for around 2 days. During the experiment, the animal was head-fixed but free to move on the wheel in the dark. Extracellular activity was recorded with glass micropipettes, which were filled with 2 M NaCl solution and advanced into the cerebellar cortex from the surface of lobule VI at an angle of 45 degrees. At the end of each experiment, brief pressure was delivered to the pipette, which was filled with Alcian Blue (0.1% solution in saline, Sigma-Aldrich, St. Louis, MO, USA) to mark the recoding site. Electrode signals were filtered, amplified and stored for off-line analyses. The locomotion activity of the animal was captured with a side camera (scA640-120 gc, Basler, Ahrensburg, DE) operating at a frame rate of 120 Hz, and the motion of the wheel was registered with an optical modular encoder (EH30M500Z5P6X3PR, Eltra, IT). Purkinje cells were identified by the occurrence of both simple spikes and complex spikes. Single-unit activity was confirmed by a pause in simple spike firing following each complex spike. To assure the quality and reliability of the recording, raw data traces with relatively stable simple spike amplitude and locomotion periods of at least 20 s were selected for analysis. Spiking activity was analysed using Spike Train (Neurasmus BV, Rotterdam, NL) and Matlab (MathWorks, Natick, MA, USA). The correlation between velocity of wheel motion and Purkinje cell activity was evaluated for each epoch of 2000 ms, which was moved through the whole movement period in every recording with increments of 200 ms. During each epoch window, the wide range of wheel velocity was standardised by *z*-score transformation, while the instantaneous firing frequency of simple spikes was smoothed by convolution with a Gaussian window of 200 ms. The correlation between wheel velocity and simple spike firing was assessed by calculating the maximum absolute value of the coefficient of Pearson correlation for each epoch. Genotypes were compared using Mann-Whitney *U* test with SPSS (IBM Corporation, Armonk, NY, USA). Data sets were analysed with the investigator blinded to the genotype of the animals.

## Results

### Mice with selective Foxp2 disruptions in the cerebellum, striatum or cortex are viable and show grossly normal development

**Fig. 1:**
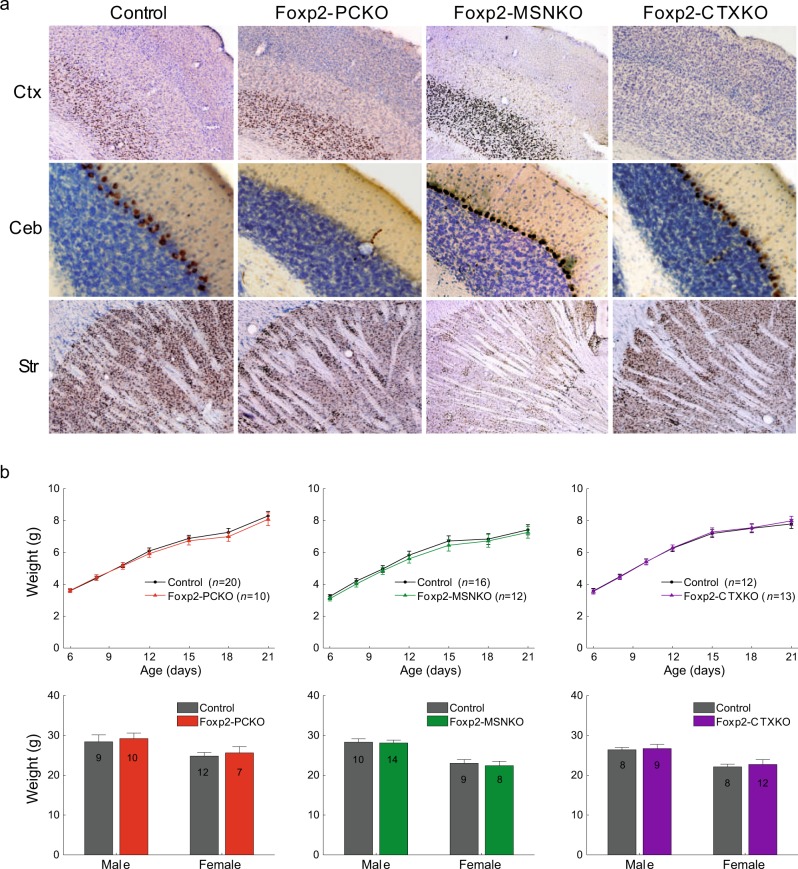
Conditional disruption of Foxp2. **a** Representative images of the cerebral cortex (Ctx), cerebellum (Ceb) and striatum (Str) taken from adult Foxp2 stained (brown) sagittal sections of conditional knockouts and a control. **b** Weights of Foxp2 conditional knockouts and controls in 6–21 day old pups (top panel) and in adult animals of experimental age (bottom panel). Animal numbers are indicated in the bars of the bottom panel. Error bars represent ± s.e.m

We disrupted Foxp2 in cerebellar Purkinje cells, striatal medium-sized spiny neurons (MSNs) or layer 5–6 neurons of the cerebral cortex by generating *L7-Cre* [34]/*Foxp2-flox/flox* [21], *Rgs9-Cre* [35]/*Foxp2-flox/flox* and *Emx1-Cre* [36]/ *Foxp2-flox/flox* mice, respectively (referred to from here on as Foxp2-PCKO, Foxp2-MSNKO and Foxp2-CTXKO). *Cre* expression begins postnatally in the *L7-Cre* and *Rgs9-Cre* lines and embryonically in the *Emx1-Cre* line (for spatiotemporal details of *Cre* expression see Materials and Methods as well as references [34–36]). Virtually no Foxp2 expression was seen in the Purkinje cells of Foxp2-PCKO mice or the cortex of Foxp2-CTXKO mice, and Foxp2 knockdown was substantial throughout the striatum of Foxp2-MSNKO mice, although some Foxp2-expressing cells were visible in more lateral areas (Fig. 1a). Importantly, Foxp2 knockdown was specific to the targeted cell types, e.g. Foxp2 expression in the cortex and striatum of Foxp2-PCKO mice was normal. All Foxp2 conditional knockouts appeared healthy and gained weight at the same rate as control littermates (Cre^-^/*Foxp2-flox/flox*) (*F*s ≤ 0.24, *ps* > 0.05; unless otherwise stated all statistics are repeated measures ANOVA effect of genotype, for full statistical analyses see Supplementary Information), and no weight differences became evident in adult animals (*t*-tests; *ts* ≤ 0.45, *ps* > 0.05) (Fig. 1b). This contrasts with the early postnatal lethality observed in Foxp2 global knockout mice [21–23]. In addition, no gross abnormalities in brain morphology were observed. There was no difference in the average number of Purkinje cells per 0.5 mm distance between Foxp2-PCKO mice and controls, and cell counts did not reveal any changes in neuron density in the striatum or layers 5–6 of the cerebral cortex in Foxp2-MSNKO and Foxp2-CTXKO animals (Supplementary Table 2). Since Foxp2 global knockouts have been reported to have a disproportionally small cerebellum [21, 22], we also examined the morphology of the cerebellum in more detail in Foxp2-PCKO mice and controls at the level of its input stage (i.e. granular layer), its integrative stage (i.e. molecular layer), and its output stage (i.e. cerebellar nuclei). We did not find obvious differences in number of granule cell dendrites, thickness of the molecular layer or density of cells in the cerebellar nuclei (Supplementary Figure S1).

### Foxp2-PCKO and Foxp2-MSNKO mice show reduced lever-press rates

**Fig. 2:**
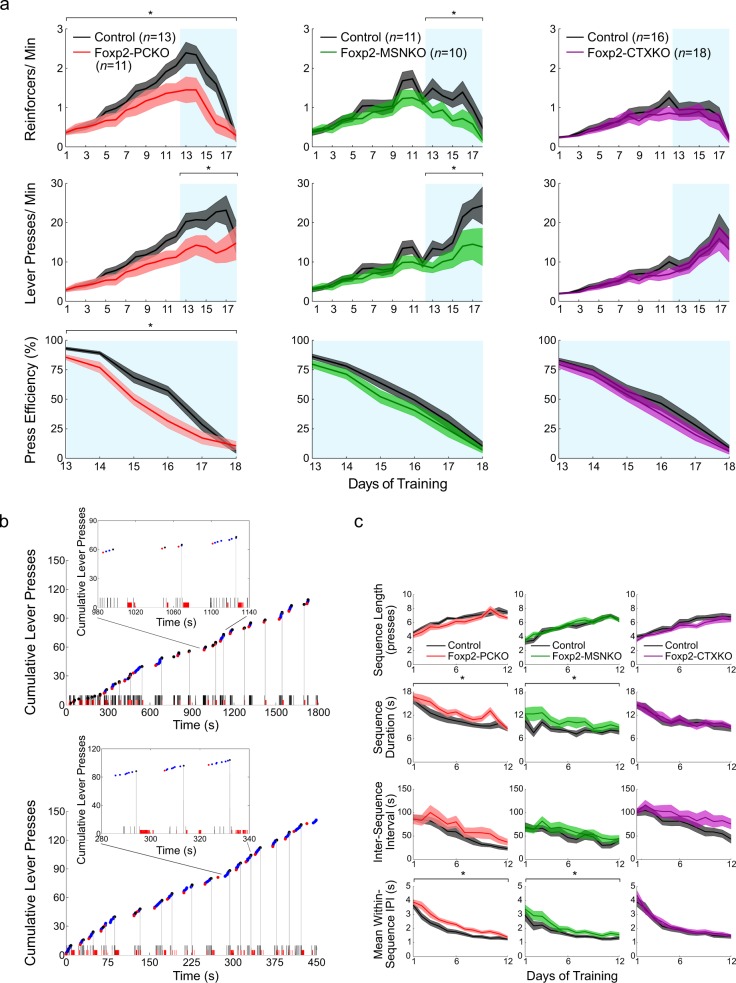
Operant lever-pressing in Foxp2 conditional knockouts. **a** Rates of reinforcer delivery (top panel) and lever pressing (middle panel) throughout the FR8 task. (Bottom panel) press efficiency during the high-speed phase. Blue shading indicates high-speed sessions. **b** Examples of the behavioural microstructure of a control animal from day 1 (top panel) and day 12 (bottom panel) of the self-paced phase of the FR8 task. Blue dots represent lever presses, with red and black dots indicating the first and last presses of sequences. Black and red ticks on the *x* axis represent head entries into the food magazine and licks in the food bowl, respectively. Grey vertical lines denote reinforcer deliveries. **c** Number of lever presses in a sequence (top panel), sequence duration (second panel), inter-sequence interval (third panel) and mean within-sequence IPI (bottom panel) during the self-paced phase of the FR8 task. Error bands represent ± s.e.m

An operant lever-pressing task was used to investigate the learning and performance of novel motor-sequences in Foxp2 conditional knockouts. In this task mice get a sucrose reinforcer after eight lever presses (fixed-ratio 8 schedule, FR8). Initially pressing is self-paced, but after 12 days of training a time constraint is added and the eight presses must be completed at increasingly high speeds (first day: eight presses in 16 s; second day: eight presses in 12 s; subsequent days: eight presses in 8, 6, 4 and 2 s). There is no signalling of the correct number of presses or the availability of the sucrose reinforcer (details in Materials and Methods). Foxp2-PCKO mice showed a reduced rate of reinforcer delivery throughout training compared to littermate controls (*F*_1,22_ = 4.48, *p* < 0.05), and a reduced rate of lever pressing during the high-speed phase (*F*_1, 22_ = 6.62, *p* < 0.05) (Fig. 2a). Furthermore, for these mice pressing was less efficient in the high-speed training phase (*F*_1,22_ = 8.59, *p* < 0.05) [press efficiency % = (reinforcers delivered x 8/lever presses) x 100]. Foxp2-MSNKO mice also showed reduced rates of reinforcer delivery and lever pressing, but only during the high-speed phase (*F*_1,29_ = 6.20, *p* < 0.05; *F*_1,19_ = 8.55, *p* < 0.05), and press efficiency was not affected (*F*_1,19_ = 2.41, *p* > 0.05). No significant differences were seen between Foxp2-CTXKO mice and littermate controls (*Fs*_1,32_ ≤ 1.23, *ps* > 0.05). Note that when examining reinforcer delivery and lever-press rates, the control groups for the three Foxp2 conditional knockout lines were significantly different from each other (*Fs*_2,37_ ≥ 6.33, *ps* < 0.05), meaning that mutant groups should not be compared directly. These differences between controls are likely to be caused by the varying genomic backgrounds of the Cre lines (details in Materials and Methods), and standard practice was followed by only directly comparing mutants with their littermates of the same genomic background. Together, these data indicate that lever-pressing behaviour is differentially affected in Foxp2 conditional knockouts.

Chunking of lever-press sequences has previously been shown to occur in the FR8 task with training, a process which is disrupted when striatal circuits are perturbed [39]. Consistent with these data, pressing in the self-paced training phase became progressively organised into discrete sequences (Fig. 2b). The number of presses in a sequence increased significantly across training in all Foxp2 conditional knockouts (effect of training day; *Fs* ≥ 27.78, *ps* < 0.001) until it was close to eight (Fig. 2c). However, it did not vary between any of the conditional knockouts and their respective controls (*Fs* ≤ 2.43, *ps* > 0.05). Instead, an increase in sequence duration and average within-sequence IPI was seen in Foxp2-PCKO (*F*_1,22_ = 4.50, *p* < 0.05; *F*_1,22_ = 8.16, *p* < 0.01) and Foxp2-MSNKO mice (*F*_1,19_ = 5.04, *p* < 0.05; *F*_1,19_ = 4.45, *p* < 0.05), likely reflecting the deficits uncovered in the initial analyses. In the high-speed training phase a similar picture emerged, with again no significant changes in sequence length between Foxp2 conditional knockouts and controls (*Fs* ≤ 2.06, *ps* > 0.05) (Supplementary Figure S2). Therefore, it appears that the lever-pressing deficits observed in Foxp2-PCKO and Foxp2-MSNKO mice are not caused by changes in the overall organisation of lever-press sequences, but rather by alterations in the timing of pressing.

### Selective Foxp2 disruptions affect the microstructure of lever-press sequences in distinct ways

**Fig. 3:**
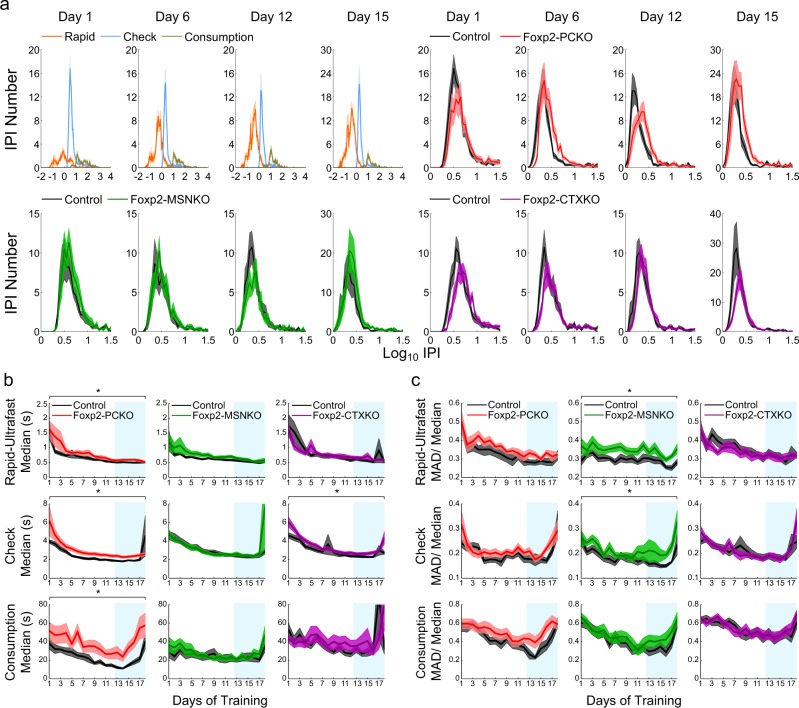
Microstructure of lever-pressing behaviour. **a** Distributions of rapid, check and consumption IPIs from Foxp2 Purkinje cell control animals at four time points during FR8 training (top left panel). Check IPI distributions of Foxp2-PCKO, Foxp2-MSNKO and Foxp2-CTXKO with controls at the same time points (top right, bottom left and bottom right panels, respectively). Average **b** median and **c** MAD/ median values of the rapid, check and consumption IPI groups of Foxp2 conditional mutants and controls during FR8 training. Blue shading indicates high-speed sessions. Error bands represent ± s.e.m

In order to look in detail at the microstructure of lever-pressing behaviour, IPIs were divided into three types: rapid (no event between presses), check (presses separated by head entry into the food magazine) and consumption (presses separated by head entry and licking) (see Materials and Methods, Fig. 3a). Each IPI type constituted a similar percentage of total IPIs in all Foxp2 conditional knockouts and controls (Supplementary Figure S3). Rapid IPI distributions were bimodal and a low point was consistently found at around 0.25 s throughout training. This value was used as a threshold to separate ultrafast from other rapid IPIs (Supplementary Figure S4a). Ultrafast IPI median values did not change across training (effect of training day; *Fs* ≤ 1.79, *ps* > 0.05), indicating that these press-sequences are not learned and may reflect bouncing of the lever (Supplementary Figure S4b). They were therefore deducted from the rapid IPI groups before conducting further analyses.

When the IPI distributions of Foxp2 conditional knockouts and their respective control groups were compared, differences became evident (Fig. 3a, Supplementary Figure S4a, Supplementary Figure S5). Foxp2-PCKO mice showed slower lever-pressing, which was reflected in increased median values of the rapid, check and consumption IPI distributions compared to controls (*F*_1,22_ = 5.66, *p* < 0.05; *F*_1,22_ = 7.08, *p* < 0.05; *F*_1,22_ = 8.09, *p* < 0.05) (Fig. 3b). In contrast, the medians of all IPI distributions were unchanged in Foxp2-MSNKO mice (*Fs* ≤ 2.91, *ps* > 0.05). In Foxp2-CTXKO mice, the check IPI median was increased (*F*_1,32_ = 5.78, *p* < 0.05). The variability of IPI distributions was assessed using the MAD/median (Fig. 3c). Notably, rapid and check IPIs were more variable in Foxp2-MSNKO mice than controls (*F*_1,19_ = 16.47, *p* < 0.05; *F*_1,19_ = 4.68, *p* < 0.05), but no differences were seen in Foxp2-PCKO or Foxp2-CTXKO mice (*Fs* ≤ 3.42, *ps* > 0.05). This increase in variability was not due to the influence of very long IPIs (i.e. when animals become disengaged from the task), because the result was the same even when only IPIs of ≤20 s were considered in the analysis (*F*_1,19_ = 15.94, *p* < 0.05; *F*_1,19_ = 5.22, *p* < 0.05) (Supplementary Figure S4c, d). In summary, Foxp2 disruption in cerebellar Purkinje cells and striatum has differential effects on the execution of lever-press sequences, with Foxp2-PCKO mice pressing more slowly and Foxp2-MSNKO mice more variably.

### Foxp2-PCKO mice show deficits in locomotor learning

The ErasmusLadder was used to investigate sequencing of locomotor movements in Foxp2 conditional knockouts. This piece of equipment consists of two parallel sets of horizontal rungs with a shelter box at each end (see Materials and Methods) [40]. A light and then an air puff encourage mice to leave the shelter boxes and they are hence trained to run back and forth across the ladder. The presence of these stimuli mean that any differences in animals’ motivation to initiate crossings would have relatively little effect on task performance [30].

**Fig. 4:**
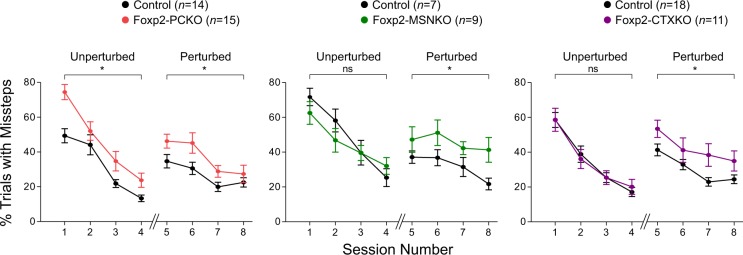
Skilled locomotion on the ErasmusLadder. Percentage trials with multiple missteps during unperturbed (days 1–4) and perturbed (days 5–8) sessions. Error bars represent ± s.e.m

Initially, even numbered rungs on one side of the ladder and odd numbered rungs on the other side were systematically elevated to generate an alternating stepping pattern. In naïve mice this condition automatically generates missteps, which is the most reliable and most sensitive parameter for motor performance [30]. In these unperturbed sessions the percentage of trials with multiple missteps (touching lower rungs two or more times) was higher in Foxp2-PCKO mice than controls (*F*_1,27_ = 8.15, *p* < 0.05), but unchanged in Foxp2-MSNKO and Foxp2-CTXKO mice (*Fs* ≤ = 0.46, *ps* > 0.05) (Fig. 4). After 4 days, perturbed sessions began and a tone was sounded 200 ms before a lower rung was elevated immediately in front of the mouse. This challenge evoked a general increase in the speed of the locomotion response, which made it more difficult for mice to place their paws accurately. Indeed, during perturbed sessions we observed an increased number of trials with multiple missteps not only in Foxp2-PCKO mice (*F*_1,27_ = 5.96, *p* < 0.05), but also in Foxp2-MSNKO and Foxp2-CTXKO mice (*F*_1,14_ = 5.35, *p* < 0.05; *F*_1,27_ = 6.38, *p* < 0.05) compared to controls. Since missteps occurred both before and after tone onset, it is unlikely that these deficits reflect a hearing problem. Calculating the total number of missteps in a session gave similar results to the multiple misstep per trial analysis, although effects were less pronounced (Supplementary Figure S6a).

Motor function was also investigated using the accelerating rotarod, the task initially used to identify motor-skill learning deficits in Foxp2 global heterozygous mutants and knockouts [22, 24]. No differences in latency to fall were observed between any of the Foxp2 conditional knockouts and their respective controls (*Fs* ≤ 0.80, *ps* > 0.05) (Supplementary Figure S6b). Taken together, the data from the operant lever-pressing, ErasmusLadder and rotarod tasks suggest that finely regulated and complex motor behaviour is affected in Foxp2 conditional knockouts, whereas gross motor behaviour is relatively normal. In addition, the advantages of more sensitive rodent tasks and associated analyses for gaining nuanced insights into gene function are highlighted.

### Increased intrinsic activity and decreased modulation of Purkinje cells in Foxp2-PCKO mice

**Fig. 5:**
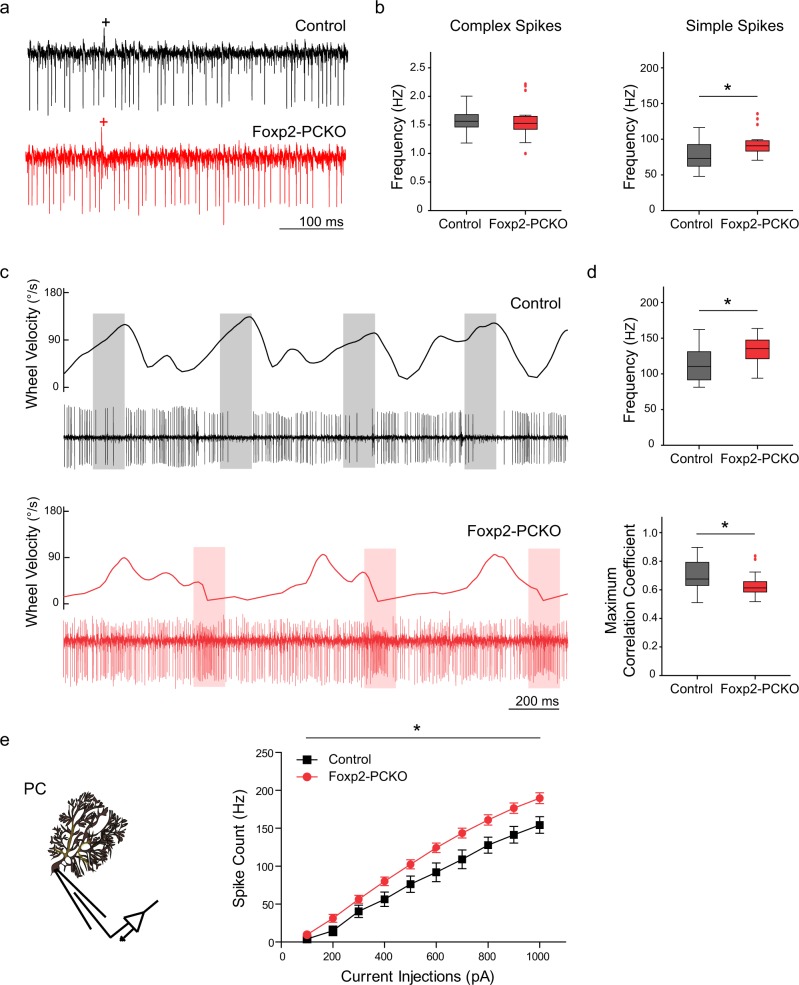
Electrophysiological recordings of Purkinje cells in control and Foxp2-PCKO mice. **a** Example traces showing simple spikes and complex spikes (+) of Purkinje cells in Foxp2-PCKO (red) and control mice (black) in vivo. **b** Increased simple spike firing (right panel) but normal complex spike activity (left panel) in Foxp2-PCKO mice at rest. **c** Example traces with wheel motion velocity plotted in the upper panels. Transparent boxes in grey indicate epochs of reduced Purkinje cell activity in relation to pre-peak velocity in control mice, whereas boxes in red indicate increased activity at trough velocity in Foxp2-PCKO mice. **d** Foxp2-PCKO mice showed higher Purkinje cell activity (upper panel) and a lower maximum correlation coefficient between wheel velocity and simple spike response (lower panel) during spontaneous locomotion. **e** Foxp2-PCKO mice showed higher output of action potentials when injected with different currents in the Purkinje cell in vitro (control *n* = 11, 3 mice; Foxp2-PCKO *n* = 11, 2 mice). Error bars represent ± s.e.m

Foxp2-PCKO mice displayed the most prominent motor-skill impairment of the three conditional knockout lines, so we investigated what electrophysiological changes might underlie these behavioural abnormalities. Effects of Foxp2 loss on in vivo Purkinje cell function were examined during limb movements. Experiments were performed in head-fixed mice while they walked on a wheel, with extracellular activity recorded from Purkinje cells in lobules III-V of the anterior lobe, an area implicated in locomotion control [44]. Locations of recording sites were confirmed by injection with Alcian blue (Supplementary Figure S7b). Purkinje cells involved in locomotion showed an increase in simple spike activity during walking compared to rest in all animals (*F*_1, 28_ = 154.79, *p* < 0.05) (Supplementary Figure S7a). However, simple spike, but not complex spike activity, was significantly higher in Foxp2-PCKO mice compared to controls at rest (Mann-Whitney *U* test; *U* = 59.00, *p* < 0.05) (Fig. 5a, b) and during locomotion (Mann-Whitney *U* test; *U* = 63.00, *p* < 0.05) (Fig. 5c, d). Furthermore, Foxp2-PCKO mice did not show characteristic epochs of reduced Purkinje cell activity when the wheel was approaching its peak velocity (Fig. 5c), and the correlation between wheel velocity and simple spike activity (see Materials and Methods) was significantly lower than that of controls (Mann-Whitney *U* test; *U* = 162.00, *p* < 0.05) (Fig. 5d). In contrast, recordings from Purkinje cells of Foxp2-MSNKO mice and controls at rest uncovered no differences in complex spike or simple spike activity between genotypes (Supplementary Figure S7c, d). This indicates that the motor impairments observed in Foxp2-MSNKO mice are not due to indirect effects of striatal Foxp2 knockdown on Purkinje cell function.

To elucidate the mechanisms underlying the abnormal in vivo modulation of simple spike firing, we investigated Purkinje cell activity in vitro using whole-cell recordings. Foxp2-PCKO and control mice showed no differences in the amplitude of excitatory postsynaptic currents recorded in Purkinje cells when parallel fibres were stimulated with increasing intensity (*F*_1,14_ = 1.30, *p* > 0.05) (Supplementary Figure S8a). Paired pulse facilitation (*F*_1,11_ = 2.99, *p* > 0.05), input resistance and holding current (*t*-tests; *ts* ≤ 0.90, *ps* > 0.05) were also normal in Foxp2-PCKO animals (Supplementary Figure S8b-d). Possible dysfunction of inhibitory input to Purkinje cells in Foxp2-PCKO mice was investigated by measuring spontaneous inhibitory postsynaptic currents, but no abnormalities were found in their frequency or amplitude (*t*-tests; *ts* ≤ 0.19, *ps* > 0.05) (Supplementary Figure S9). Importantly, when depolarising currents were injected at the cell soma, Purkinje cells in Foxp2-PCKO animals exhibited significantly higher intrinsic excitability than controls (*F*_1,20_ = 5.97, *p* < 0.05) (Fig. 5e). However, the action potentials generated by current injection were not different in amplitude, threshold, after-hyperpolarization or half-width (t-tests; *ts*_20_ ≤ 1.35, *ps* > 0.05) (Supplementary Figure S10). Together, these findings suggest that Foxp2 perturbation may affect the expression level of subthreshold conductances, which in turn may increase simple spike firing, while the action potential generation machinery is intact.

## Discussion

In this study we used a conditional approach to selectively remove Foxp2 from cerebellar Purkinje cells, striatal medium-sized spiny neurons or layer 5–6 cells of the cerebral cortex. These are key Foxp2-expressing areas that are also known to be involved in motor-sequence learning, and their importance has been emphasised in previous studies of this gene in humans, mice and songbirds. We subjected each of our Foxp2 conditional knockout lines to behavioural tasks to investigate their motor-sequence learning and performance in depth. Foxp2 disruption in Purkinje cells led to prominent deficits. Foxp2-PCKO animals executed press-sequences of all types more slowly than controls, and showed limb placement deficits during both unperturbed and perturbed sessions on the ErasmusLadder. In vivo recordings from Purkinje cells of Foxp2-PCKO mice uncovered an increase in simple spike activity during both rest and locomotion and a reduced correlation between wheel velocity and simple spike activity. Furthermore, whole-cell recordings showed that these changes are likely, at least in part, to be caused by an increase in the intrinsic excitability of Purkinje cells.

The increase in firing rate and the reduction in correlation with motor output are in line with a recent hypothesis on the learning mechanisms in zebrin-negative modules of the cerebellum [45, 46]. In the zebrin-negative modules, which incorporate locomotion control regions [44], simple spike firing frequency is usually suppressed during learning [47], as opposed to the zebrin-positive zones where firing frequency is increased [45]. If the intrinsic excitability is too high, the firing frequency may also remain too high in zebrin-negative modules, reducing the modulation magnitude and correlation with motor output [48–50], hence preventing an adequate build-up of an internal model [51]. How the transcription factor Foxp2 exerts its impact on intrinsic excitability of Purkinje cells is beyond the scope of the current study, but one might speculate that factors like those of the G-protein coupled receptor protein signalling pathway, which have been shown to depend on Foxp2 [11], contribute to the excitability of Purkinje cells by affecting the efficacy of SK channels [52, 53]. Another candidate which may affect the regulation of ion-conductances in Purkinje cells is Cntnap2 (contactin-associated protein-like-2), a transmembrane protein belonging to the neurexin family [54]. Cntnap2is a direct downstream target of Foxp2 [55], and is involved in the modulation of Kv1.2 channels [56]. Interestingly, Cntnap2 has been shown to affect Pavlovian eyeblink conditioning [57], which is another typical cerebellar form of procedural memory formation controlled by a zebrin-negative module [45]. Thus, there are various ways by which the absence of Foxp2 in Purkinje cells could reduce suppression of simple spikes and thereby increase the excitability of these cells.

The deficits seen in Foxp2-MSNKO mice were distinct from those seen in Foxp2-PCKO mice. Interestingly, Foxp2-MSNKO mice executed rapid and check press-sequences more variably. This finding is consistent with independent data from zebra finches, where FoxP2 knockdown in area X of the striatum in juvenile or adult birds increases variability of the syllables that comprise song [27]. Plasticity of striatal inputs is thought to be necessary for crystallising new motor-skills, and disrupting plasticity in the striatum leads to more variable behaviour in mice [39]. Striatal plasticity is aberrantly modulated in mice heterozygous for the KE-family mutation [24], and although we do not know if this is the case in Foxp2-MSNKO mice, it suggests a potential mechanism that could account for the increased behavioural variability that we observe in these animals.

Dopamine is implicated in modulating the variability of learned motor behaviour in basal-ganglia circuits [58, 59]. In zebra finch the dopamine levels of area X are elevated during directed (less variable) relative to undirected (more variable) singing [60], and infusion of a dopamine receptor 1 (D1R) antagonist can abolish these context-dependent changes in song [61]. FoxP2 knockdown in area X also disrupts the regulation of song variability by social context, and interferes with D1R-mediated modulation of activity propagation through the anterior forebrain pathway, possibly by down-regulating expression of D1R and DARPP-32 [27]. Consistently, Foxp2 knockdown in area X also reduces the density of dendritic spines on MSNs [62], where many D1Rs are located [63]. In mice, Foxp2 is preferentially expressed in D1R compared to D2R MSNs [11, 64] and global heterozygous Foxp2 knockouts also have altered dopamine levels in the brain [65]. Thus, it will be interesting to find out to what extent changes in the efficacy of dopamine-dependent processing in cortico-striatal circuitries also contributes to the more variable behaviour seen in Foxp2-MSNKO mice [66].

Foxp2-CTXKO mice were characterised by relatively subtle deficits and showed slower check IPIs as well as increased numbers of trials with multiple missteps during perturbed sessions on the ErasmusLadder. Since Foxp2-CTXKO mice were generated using the *Emx1-Cre* line [36], which is also expressed in the dorsal spinal cord, we cannot exclude the possibility that motor neuron development is affected in these animals. However, it should be noted that Foxp2-CTXKO mice showed virtually no impairment in baseline motor performance (e.g. during unperturbed sessions on the ErasmusLadder), so it is unlikely that deficits resulting from impairment of motor neuron function act as a severe confound. The main learning deficits in Foxp2-CTXKO mice are in fact more in line with dysfunctions of higher order regions such as the cerebral cortex [67].

All three Foxp2 conditional knockout lines were able to learn and perform the motor-skills required for the behavioural tasks, albeit with significant impairment. This observation is consistent with the cerebellar learning still seen following cell-type specific deficits in the cerebellar cortex [48, 68]. Most likely, this remnant capacity for learning is partly mediated by downstream regions, such as the cerebellar and vestibular nuclei, which can compensate functionally [69, 70]. These types of mechanisms are more important when manipulations or genetic disruptions occur early in development.

Selective disruptions of Foxp2 were used to determine its functions in specific brain regions and cell types with a particular emphasis on motor-skill learning. Effects on motor-sequence learning and performance were assessed using several tasks which allowed fine resolution of motor behaviour. Mice exhibit at best limited vocal learning [71], so vocalisation behaviours were not a focus of the present study. This approach enabled us to successfully identify distinct roles for Foxp2 in motor function in Purkinje cells, striatum and cerebellar cortex. The Foxp2 conditional knockout lines showed no difference from their respective controls in the average number of lever presses in a sequence. These observations suggest that sequence structure and organisation are largely unaffected by loss of Foxp2 from the targeted brain regions, although deficits could become evident with more complex heterogeneous sequences. Differences between knockouts and controls emerged only after analyses of the temporal microstructure of lever pressing, with distinct effects depending on the site of Foxp2 loss. Foxp2-PCKO mice executed all press-sequences more slowly, whereas Foxp2-MSNKO mice executed rapid and check press-sequences more variably. Consistently, a greater number of missteps were seen in Foxp2-PCKO mice during both unperturbed and perturbed conditions on the ErasmusLadder, whilst deficits in Foxp2-CTXKO and Foxp2-MSNKO were seen only in perturbed conditions. Hence, the most striking effects were seen in mice with disruption of Foxp2 specifically in Purkinje cells of the cerebellum, a region known to be involved in learning timing-sensitive processes [30, 44, 72]. Indeed, similar impairments on the ErasmusLadder have been seen in several other mouse lines with cell-type specific disruptions of cerebellar proteins [30]. In humans, individuals with cerebellar lesions show impaired sequence learning on a serial reaction time task [73], whereas basal ganglia lesions result in more subtle deficits in speed and force control of finger tapping [74].

The majority of brain lesions causing verbal dyspraxia occur in cortical and sub-cortical regions, and most functional FoxP2 work to date has focused on cortico-striatal circuits. Nevertheless, it is becoming increasingly clear that the role of the cerebellum goes beyond that of a coordinator of basic motor function [75] and extends to regulating purposeful skilled motor actions such as those required for spoken language [76]. Indeed, in line with the finding that cerebellar lesions can give rise to verbal dyspraxia by disrupting cerebro-cerebellar connectivity [77], functional imaging has shown altered activity in both the cerebral cortex and the cerebellum of affected KE family members during language tasks [17, 78]. Here we identify a role for Foxp2 in Purkinje cells in regulating skilled motor behaviour in mice and provide a mechanism by which this could occur. This function could potentially be relevant for fine motor skills such as speech, and our data highlight the need to consider cerebellar circuits together with cortico-striatal circuits when investigating Foxp2-associated disorder.

## Electronic supplementary material

Supplementary Information

Supplementary Table 1

Supplementary Table 2

Supplementary Figure 1

Supplementary Figure 2

Supplementary Figure 3

Supplementary Figure 4

Supplementary Figure 5

Supplementary Figure 6

Supplementary Figure 7

Supplementary Figure 8

Supplementary Figure 9

Supplementary Figure 10
