# Supplementary material for: Differential effects of Foxp2 disruption in distinct motor circuits
Source: Mol Psychiatry. 2018 Aug 14;24(3):447–62. doi: 10.1038/s41380-018-0199-x (PMC6514880; doi:10.1038/s41380-018-0199-x)
Supplement: Supplementary file 1 — Supplementary Information [file 41380_2018_199_MOESM1_ESM.docx]

**Supplementary Information**

**Differential effects of Foxp2 disruption in distinct motor circuits**

Catherine A. French, María F. Vinueza Veloz, Kuikui Zhou, Saša Peter, Simon E. Fisher, Rui M. Costa and Chris I. De Zeeuw

**Supplementary Figure 1. The morphology of granule cells which provide input to Purkinje cells and that of cerebellar nuclei neurons which receive input from Purkinje cells appears normal in Foxp2-PCKO mice. (Top panel)** Examples of Golgi stained granule cells from control animals (left) and Foxp2-PCKO animals (right). The average number of dendrites per granule cell was not significantly different (*p* > .05; Mann-Whitney) between controls (3.50 ± 0.14, *n* = 2) and Foxp2-PCKO mice (3.40 ± 0.30, *n* = 2). **(Bottom panel)** Representative cerebellar sections immunostained for Foxp2 from a control animal (left) and a Foxp2-PCKO mouse (right) (arrows show examples of labelled cells in DN). Note that in controls all Purkinje cells and some cerebellar nuclei neurons are densely stained for Foxp2 (left), whereas in Foxp2-PCKO animals only cerebellar nuclei neurons are labelled (right). Cerebellar nuclei cells expressing Foxp2 were counted using Neurolucida software in 3-4 representative sections per animal. The average density of labelled cells was not significantly different (*p* > .05; Mann-Whitney) between control (512 ± 58 cells/ mm^2^, *n* = 3) and Foxp2-PCKO mice (492 ± 48 cells/ mm^2^, *n* = 3). The thickness of the molecular layer was also measured and no difference was found between genotypes (*p* > 0.5; Mann-Whitney) (controls: 2.05 ± 0.25 mm^2^, *n* = 3 and Foxp2-PCKO: 2.01 ± 0.12 mm^2^, *n* = 3). Abbreviations: DN = dentate nucleus; DCN = dorsal cochlear nucleus; Ceb Ctx = cerebellar cortex.

**Supplementary Figure 2. Operant lever-pressing in Foxp2 conditional knockouts during the high-speed training phase. (a)** Examples of the behavioural microstructure of a control animal from the high-speed phase of the FR8 task. Mice must presses 8 times in 16 s (top panel) or 6s (bottom panel). Blue dots represent lever presses, with red and black dots indicating the first and last presses of sequences. Black and red ticks on the x axis represent head entries into the food magazine and licks in the food bowl respectively. Grey vertical lines denote reinforcer deliveries. **(b)** Number of lever presses in a sequence (top panel), sequence duration (second panel), inter-sequence interval (third panel) and mean within-sequence IPI (bottom panel) during the high-speed phase of the FR8 task. Sequence length increased as the task became progressively harder, but there were no differences between Foxp2 conditional knockouts and controls. Increases in inter-sequence interval and mean within-sequence IPI were seen in Foxp2-PCKO mice with the former also elevated in Foxp2-MSNKO mice. Error bands represent + s.e.m.

Statistical analyses:

*Sequence length:*

Foxp2-PCKO repeated measures ANOVA (square root data transformation): Genotype [*F*_1, 22_ = 2.06, *p* > .05]; Day [*F*_1.83, 40.25_ = 14.19, *p* < .001]; Interaction [*F*_1.83, 40.25_ = 0.95, *p* > .05].

Foxp2-MSNKO repeated measures ANOVA (square root data transformation): Genotype [*F*_1, 19_ = 2.01, *p* > .05]; Day [*F*_1.60, 30.38_ = 17.06, *p* < .001]; Interaction [*F*_1.60, 30.38_ = 0.70, *p* > .05].

Foxp2-CTXKO repeated measures ANOVA (untransformed): Genotype [*F*_1, 32_ = 1.52, *p* > .05]; Day [*F*_2.81, 90.03_ = 30.99, *p* < .001]; Interaction [*F*_2.81, 90.03_ = 2.43, *p* > .05].

*Sequence duration:*

Foxp2-PCKO repeated measures ANOVA (natural log data transformation): Genotype [*F*_1, 22_ = 2.76, *p* > .05]; Day [*F*_2.45, 53.86_ = 10.51, *p* < .001]; Interaction [*F*_2.45, 53.86_ = 1.57, *p* > .05].

Foxp2-MSNKO repeated measures ANOVA (natural log data transformation): Genotype [*F*_1, 19_ = 0.08, *p* > .05]; Day [*F*_2.03, 38.57_ = 10.72, *p* < .001]; Interaction [*F*_2.03, 38.57_ = 0.57, *p* > .05].

Foxp2-CTXKO repeated measures ANOVA (natural log data transformation): Genotype [*F*_1, 32_ = 0.38, *p* > .05]; Day [*F*_2.90, 92.80_ = 13.85, *p* < .001]; Interaction [*F*_2.90, 92.80_ = 1.99, *p* > .05].

*Inter-sequence interval:*

Foxp2-PCKO repeated measures ANOVA (natural log data transformation): Genotype [*F*_1, 22_ = 7.54, *p* < .05]; Day [*F*_3.47, 76.39_ = 15.48, *p* < .001]; Interaction [*F*_3.47, 76.39_ = 1.29, *p* > .05].

Fisher’s LSD post hocs:

| 16s | 12s | 8s | 6s | 4s | 2s |
| --- | --- | --- | --- | --- | --- |
| *p* < .05 | *p* < .05 | *p* < .05 | *p* < .05 | *p* < .05 | ns |

Foxp2-MSNKO repeated measures ANOVA (natural log data transformation): Genotype [*F*_1, 19_ = 6.57, *p* < .05]; Day [F_2.86, 54.24_ = 2.21, *p* > .05]; Interaction [*F*_2.86, 54.24_ = 0.24, *p* > .05].

Fisher’s LSD post hocs:

| 16s | 12s | 8s | 6s | 4s | 2s |
| --- | --- | --- | --- | --- | --- |
| ns | ns | ns | ns | *p* < .05 | *p* < .05 |

Foxp2-CTXKO repeated measures ANOVA (natural log data transformation): Genotype [*F*_1, 32_ = 0.44, *p* > .05]; Day [*F*_3.75, 120.08_ = 2.78, *p* < .05]; Interaction [*F*_3.75, 120.08_ = 0.19, *p* > .05].

*Within-sequence IPI:*

Foxp2-PCKO repeated measures ANOVA (untransformed): Genotype [*F*_1, 22_ = 4.40, *p* < .05]; Day [*F*_3.42, 75.16_ = 11.37, *p* < .001]; Interaction [*F*_3.42, 75.16_ = 1.91, *p* > .05].

Fisher’s LSD post hocs:

| 16s | 12s | 8s | 6s | 4s | 2s |
| --- | --- | --- | --- | --- | --- |
| *p* < .05 | *p* < .05 | ns | *p* < .05 | ns | ns |

Foxp2-MSNKO repeated measures ANOVA (natural log data transformation): Genotype [*F*_1, 19_ = 2.60, *p* > .05]; Day [*F*_3.33, 63.31_ = 20.70, *p* < .001]; Interaction [*F*_3.33, 63.31_ = 1.22, *p* > .05].

Foxp2-CTXKO repeated measures ANOVA (natural log data transformation): Genotype [*F*_1, 32_ = 0.88, *p* > .05]; Day [*F*_2.60, 83.09_ = 14.15, *p* < .001]; Interaction [*F*_2.60, 83.09_ = 0.35, *p* > .05].

**Supplementary Figure 3. Proportion of presses in each of the three inter-press interval groups.** Percentage of total IPIs in the rapid, check and consumption groups of Foxp2 conditional knockouts and controls during FR8 training. Blue shading indicates high-speed sessions. The proportion of IPIs classed as rapid increased as training progressed and a corresponding decrease was seen in check and consumption IPIs. However, there were no differences between Foxp2 conditional knockouts and controls, with the exception of a small increase in the proportion consumption IPIs in Foxp2-PCKO mice. Error bands represent + s.e.m.

Statistical analyses:

*Rapid:*

Foxp2-PCKO repeated measures ANOVA (untransformed): Genotype [*F*_1, 22_ = 1.18, *p* > .05]; Day [*F*_4.84, 106.51_ = 57.56, *p* < .001]; Interaction [*F*_4.84, 106.51_ = 1.51, *p* > .05].

Foxp2-MSNKO repeated measures ANOVA (square data transformation): Genotype [*F*_1, 19_ = 0.16, *p* > .05]; Day [*F*_4.78, 90.77_ = 75.90, *p* < .001]; Interaction [*F*_4.78, 90.77_ = 1.24, *p* > .05].

Foxp2-CTXKO repeated measures ANOVA (square data transformation): Genotype [*F*_1, 32_ = 0.06, *p* > .05]; Day [*F*_5.60, 179.34_ = 77.38, *p* < .001]; Interaction [*F*_5.60, 179.34_ = 0.73, *p* > .05].

*Check:*

Foxp2-PCKO repeated measures ANOVA (untransformed): Genotype [*F*_1, 22_ = 0.38, *p* > .05]; Day [*F*_4.70, 103.36_ = 32.70, *p* < .001]; Interaction [*F*_4.70, 103.36_ = 1.35, *p* > .05].

Foxp2-MSNKO repeated measures ANOVA (untransformed): Genotype [*F*_1, 19_ = 0.44, *p* > .05]; Day [*F*_4.57, 86.82_ = 15.26, *p* < .001]; Interaction [*F*_4.57, 86.82 =_ 1.72, *p* > .05].

Foxp2-CTXKO repeated measures ANOVA (square root data transformation): Genotype [*F*_1, 32_ = 0.28, *p* > .05]; Day [*F*_5.67, 181.42_ = 27.70, *p* <.001]; Interaction [*F*_5.67, 181.42_ = 0.41, *p* >.05].

*Consumption:*

Foxp2-PCKO repeated measures ANOVA (square root data transformation): Genotype [*F*_1, 22_ = 5.24, *p* < .05]; Day [*F*_4.25, 93.38_ = 48.86, *p* < .001]; Interaction [*F*_4.25, 93.38_ = 1.58, *p* > .05].

Fisher’s LSD post hocs:

| 1 | 2 | 3 | 4 | 5 | 6 | 7 | 8 | 9 | 10 | 11 | 12 | 16s | 12s | 8s | 6s | 4s | 2s |
| --- | --- | --- | --- | --- | --- | --- | --- | --- | --- | --- | --- | --- | --- | --- | --- | --- | --- |
| *p* < .05 | *p* < .05 | *p* < .05 | *p* < .05 | ns | ns | ns | ns | ns | ns | ns | ns | ns | ns | ns | ns | *p* < .05 | ns |

Foxp2-MSNKO repeated measures ANOVA (natural log data transformation): Genotype [*F*_1, 19_ = 0.74, *p* > .05]; Day [*F*_4.75, 90.29_ = 110.97, *p* < .001]; Interaction [*F*_4.75, 90.29_ = 0.54, *p* > .05].

Foxp2-CTXKO repeated measures ANOVA (square root log data transformation): Genotype [*F*_1, 32_ = 1.29, *p* > .05]; Day [*F*_7.10, 227.25_ = 84.16, *p* < .001]; Interaction [*F*_7.10, 227.25_ = 0.88, *p* > .05].

**Supplementary Figure 4. Analyses of rapid lever-pressing. (a)** Distribution of rapid IPIs from Foxp2 conditional knockouts and controls at four time points during FR8 training. The vertical black line at 0.25 s thresholds ultrafast IPIs. **(b)** Average median (top panel) and MAD/ median (middle panel) values of ultrafast IPIs in Foxp2 conditional knockouts and controls during FR8 training. Percentage of rapid IPIs that are ultrafast (bottom panel). Average MAD/ median values of **(c)** check IPIs and **(d)** rapid IPIs after ultrafast IPIs were removed in Foxp2-MSNKO mice and controls. Blue shading indicates high-speed sessions. Error bands represent + s.e.m.

Statistical analyses:

*Ultrafast IPIs - median:*

Foxp2-PCKO repeated measures ANOVA (untransformed data): Genotype [*F*_1, 22_ = 0.04, *p* > .05]; Day [*F*_8.36, 183.97_ = 1.79, *p* > .05]; Interaction [*F*_8.36, 183.97_ = 1.56, *p* > .05].

Foxp2-MSNKO repeated measures ANOVA (squared transformation): Genotype [*F*_1, 19_ = 2.19, *p* > .05]; Day [*F*_6.16, 116.95_ = 1.10, *p* > .05]; Interaction [*F*_6.16, 116.95_ = 0.84, *p* > .05].

Foxp2-CTXKO repeated measures ANOVA (untransformed data): Genotype [*F*_1, 32_ = 0.89, *p* > .05]; Day [*F*_6.93, 221.79_ = 1.65, *p* > .05]; Interaction [*F*_6.93, 221.79_ = 0.80, *p* > .05].

*Ultrafast IPIs – MAD/M:*

Foxp2-PCKO repeated measures ANOVA (untransformed data): Genotype [*F*_1, 22_ = 0.40, *p* > .05]; Day [*F*_7.45, 163.87_ = 2.55, *p* < .05]; Interaction [*F*_7.45, 163.87_ = 2.30, *p* < .05].

Foxp2-MSNKO repeated measures ANOVA (untransformed data): Genotype [*F*_1, 19_ = 0.09, *p* > .05]; Day [*F*_7.55, 143.42_ = 1.51, *p* > .05]; Interaction [*F*_7.55, 143.42_ = 1.43, *p* > .05].

Foxp2-CTXKO repeated measures ANOVA (reciprocal data transformation): Genotype [*F*_1, 32_ = 0.02, *p* > .05]; Day [*F*_9.12, 291.97_ = 2.90, *p* < .01]; Interaction [*F*_9.12, 291.97_ = 0.63, *p* > .05].

*% ultrafast IPIs:*

Foxp2-PCKO repeated measures ANOVA (square root data transformation): Genotype [*F*_1, 22_ = 1.04, *p* > .05]; Day [*F*_2.76, 60.70_ = 17.46, *p* < .001]; Interaction [*F*_2.76, 60.70_ = 0.90, *p* > .05].

Foxp2-MSNKO repeated measures ANOVA (square root transformation): Genotype [*F*_1, 19_ = 4.47, *p* < .05]; Day [*F*_3.34, 63.37_ = 9.33, *p* < .001]; Interaction [*F*_3.34, 63.37_ = 0.89, *p* > .05].

Fisher’s LSD post hocs:

| 1 | 2 | 3 | 4 | 5 | 6 | 7 | 8 | 9 | 10 | 11 | 12 | 16s | 12s | 8s | 6s | 4s | 2s |
| --- | --- | --- | --- | --- | --- | --- | --- | --- | --- | --- | --- | --- | --- | --- | --- | --- | --- |
| ns | ns | ns | *p* < .05 | ns | ns | *p* < .05 | *p* < .05 | *p* < .05 | ns | *p* < .05 | *p* < .05 | *p* < .05 | ns | ns | ns | ns | ns |

Foxp2-CTXKO repeated measures ANOVA (square root data transformation): Genotype [*F*_1, 32_ = 4.81, *p* < .05]; Day [*F*_4.98, 159.34_ = 11.53, *p* < .001]; Interaction [*F*_4.98, 159.34_ = 0.65, *p* > .05].

Fisher’s LSD post hocs:

| 1 | 2 | 3 | 4 | 5 | 6 | 7 | 8 | 9 | 10 | 11 | 12 | 16s | 12s | 8s | 6s | 4s | 2s |
| --- | --- | --- | --- | --- | --- | --- | --- | --- | --- | --- | --- | --- | --- | --- | --- | --- | --- |
| ns | ns | ns | ns | *p* < .05 | ns | ns | ns | ns | ns | ns | *p* < .05 | ns | ns | *p* < .05 | *p* < .05 | *p* < .05 | *p* < .05 |

*Rapid - ultrafast (20 s cut off) – MAD/ median:*

Foxp2-MSNKO FR8(1)-FR8(2s) repeated measures ANOVA (natural log data transformation): Genotype [*F*_1, 19_ = 15.94, *p* = .001]; Day [*F*_6.34, 120.38_ = 2.00, *p* > .05]; Interaction [*F*_6.34, 120.38_ = 0.41, *p* > .05].

Fisher’s LSD post hocs:

| 1 | 2 | 3 | 4 | 5 | 6 | 7 | 8 | 9 | 10 | 11 | 12 | 16s | 12s | 8s | 6s | 4s | 2s |
| --- | --- | --- | --- | --- | --- | --- | --- | --- | --- | --- | --- | --- | --- | --- | --- | --- | --- |
| *p* < .05 | ns | ns | ns | ns | ns | *p* < .05 | *p* < .05 | ns | ns | ns | *p* < .05 | ns | ns | ns | ns | *p* < .05 | *p* < .05 |

*Check (20 s cut off) – MAD/ median:*

Foxp2-MSNKO FR8(1)-FR8(2s) repeated measures ANOVA (natural log data transformation): Genotype [*F*_1, 19_ = 5.22, *p* < .05]; Day [*F*_6.24, 118.56_ = 3.72, *p* < .01]; Interaction [*F*_6.24, 118.56_ = 0.48, *p* > .05].

Fisher’s LSD post hocs:

| 1 | 2 | 3 | 4 | 5 | 6 | 7 | 8 | 9 | 10 | 11 | 12 | 16s | 12s | 8s | 6s | 4s | 2s |
| --- | --- | --- | --- | --- | --- | --- | --- | --- | --- | --- | --- | --- | --- | --- | --- | --- | --- |
| ns | ns | ns | ns | ns | *p* < .05 | ns | ns | ns | ns | ns | ns | ns | ns | ns | ns | ns | ns |

**Supplementary Figure 5**. **Distribution of consumption IPIs.** Consumption IPIs of Foxp2 conditional knockouts and controls at four time points during FR8 training. Error bands represent + s.e.m.

**Supplementary Figure 6. Motor-skill learning on the ErasmusLadder and the accelerating rotarod. (a)** Percentage missteps during unperturbed (days 1-4) and perturbed (days 5-8) sessions on the ErasmusLadder. **(b)** Latency to fall from a rotarod accelerating from 6-60 rpm over a 5 min period. Mice received 10 trials per day for 5 consecutive days. Error bars represent + s.e.m.

Statistical analysis:

*ErasmusLadder:*

*Foxp2-PCKO:*

Repeated measures ANOVA session 1-8: Genotype [*F*_1, 27_ = 11.44, *p* < .01]; Session [*F*_5, 123_ = 8.95, *p* < .001]; Interaction [*F*_5, 123_ = 0.46, *p* > .05].

Repeated measures ANOVA session 1-4: Genotype [*F*_1, 27_ = 11.48, *p* < .001]; Session [*F*_3, 81_ = 3.59, *p* < .05]; Interaction [*F*_3, 81_ = 0.43, *p* > .05].

Repeated measures ANOVA session 5-8: Genotype [*F*_1, 27_ = 3.99, *p* > .05]; Session [*F*_3, 81_ = 2.02, *p* > .05]; Interaction [*F*_3, 81_ = 0.20, *p* > .05].

*Foxp2-MSNKO:*

Repeated measures ANOVA session 1-8: Genotype [*F*_1, 14_ = 2.59, *p* > .05]; Session [*F*_3, 43_ = 5.33, *p* < .001]; Interaction [*F*_3, 43_ = 1.07, *p* > .05].

Repeated measures ANOVA session 1-4: Genotype [*F*_1, 14_ = 0.60, *p* > .05]; Session [*F*_3, 42_ = 11.41, *p* < .001]; Interaction [*F*_3, 42_ = 0.77, *p* > .05].

Repeated measures ANOVA session 5-8: Genotype [*F*_1, 14_ = 4.05, *p* > .05]; Session [*F*_3, 42_ = 2.05, *p* > .05]; Interaction [*F*_3, 42_ = 0.09, *p* > .05].

*Foxp2-CTXKO:*

Repeated measures ANOVA session 1-8: Genotype [*F*_1, 27_ = 0.28, *p* > .05]; Session [*F*_4, 101_ = 21.05, *p* < .001]; Interaction [*F*_4, 101_ = 1.24, *p* > .05].

Repeated measures ANOVA session 1-4: Genotype [*F*_1, 27_ = 0.47, *p* > .05]; Session [*F*_3, 81_ = 4.25, *p* < .01]; Interaction [*F*_3, 81_ = 0.46, *p* > .05].

Repeated measures ANOVA session 5-8: Genotype [*F*_1, 27_ = 2.39, *p* > .05]; Session [*F*_2, 50_ = 1.83, *p* > .05]; Interaction [*F*_2, 50_ = 1.03, *p* > .05].

*Rotarod:*

Foxp2-PCKO repeated measures ANOVA: Genotype [*F*_1, 27_ = 0.80, *p* > .05]; Day [*F*_9.11, 245.86_ = 4.80, *p* < .001]; Interaction [*F*_9.11, 245.86_ = 1.05, *p* > .05].

Foxp2-MSNKO repeated measures ANOVA: Genotype [*F*_1, 18_ = 0.39, *p* > .05]; Day [*F*_9.78, 175.95_ = 4.25, *p* < .001]; Interaction [*F*_9.78, 175.95_ = 0.75, *p* > .05].

Foxp2-CTXKO repeated measures ANOVA: Genotype [*F*_1, 23_ = 0.00, *p* > 0.05]; Day [*F*_11.10, 255.33_ = 5.90, *p* < .001]; Interaction [*F*_11.10, 255.33_ = 0.90, *p* > .05].

**Supplementary Figure 7.** **Extracellular single-unit recordings of Purkinje cells. (a)** Increased simple spike activity during locomotion compared to rest in control and Foxp2-PCKO mice (*n* = 15 for both genotypes). **(b)** Locations of recording sites were confirmed by injection with Alcian blue, which was visualised against a neutral red stained background. No differences in the firing frequency of **(c)** complex spikes or **(d)** simple spikes was seen in Foxp2-MSNKO mice compared to controls at rest (Foxp2-MSNKO: *n* = 15; control: *n* = 18).

Statistical analysis:

*Simple spike frequency:*

Foxp2-PCKO simple spike frequency repeated measures ANOVA (natural log data transformation): Genotype [*F*_1, 28_ = 4.00, *p* < .05]; Movement [*F*_1, 28_ = 154.79, *p* < .001]; Interaction [*F*_1, 28_= 0.96, *p* > .05].

*Complex spike frequency at rest:*

Foxp2-MSNKO complex spike frequency at rest Mann-Whitney U test: *U* = 170.00, *p* > .05.

*Simple spike frequency at rest:*

Foxp2-MSNKO simple spike frequency at rest Mann-Whitney U test: *U* = 175.00, *p* > .05.

**Supplementary Figure 8. PF to PC synaptic baseline input is normal in Foxp2-PCKO mice. (a)** No difference in the parallel fibre (PF) to PC input with increasing stimulation intensity was observed in excitatory postsynaptic currents (EPSCs) amplitude (control : *n* = 9 ; 3 mice Foxp2-PCKO ; *n* = 8, 2 mice) and **(b)** paired pulse facilitation (PPF) (control: *n* = 9, 3 mice; Foxp2-PCKO: *n* = 6, 2 mice), where **(c)** input resistance (Ri) (control: *n* = 9, 3 mice; Foxp2-PCKO: *n* = 8, 2 mice) and **(d)** holding current (HC) (control: *n* = 9, 3 mice; Foxp2-PCKO: *n* = 10, 2 mice) were also unaltered. Error bars represent + s.e.m.

Statistical analysis:

Stimulation strength repeated measures ANOVA Genotype [*F*_1, 14_ = 1.30, *p* > .05]. Paired pulse facilitation repeated measures ANOVA Genotype [*F*_1, 11_ = 2.99, *p* > .05]. Input resistance t-test [*t*_15_=0.66, *p* > .05]. Holding current t-test [*t*_17_=0.90, *p* > .05]

**Supplementary Figure 9. Inhibition at the PF to PC synapse is not impaired in Foxp2-PCKO mice. (a)** No difference in the inhibitory response of PCs when measuring the frequency of spontaneous inhibitory postsynaptic currents (sIPSCs) (control: *n* = 13; Foxp2-PCKO: *n* = 12, 2 mice) **(b)** and their amplitudes (control: *n* = 11; Foxp2-PCKO: *n* = 12, 2 mice). Error bars represent + s.e.m.

Statistical analysis:

Frequency of sIPSCs t-test [*t*_23_=0.19, *p* > .05]. Amplitude of sIPSCs t-test [*t*_21_=0.05, *p* > .05].

**Supplementary Figure 10. Action potentials generated in PCs of Foxp2-PCKO mice appear normal. (a)** No difference in the action potential (AP) amplitude **(b)** threshold, **(c)** after hyperpolarization and **(d)** AP half-width of control and Foxp2-PCKO mice. (control: *n* = 11, 3 mice; Foxp2-PCKO: *n* = 11, 2 mice). Error bars represent + s.e.m.

Statistical analysis:

Action potential amplitude t-test [*t*_20_=0.88, *p* > .05] threshold t-test [*t*_20_=1.35, *p* > .05], after hyperpolarization t-test [*t*_20_=0.30, *p* > .05] and action potential half-width [*t*_20_=1.32, *p* > .05].

**Full Statistical Analyses for Main Figures**

**Figure 1**

**(b) Pup weights**

Foxp2-PCKO repeated measures ANOVA (untransformed): Genotype [*F*_1, 28_ = 0.14, *p* > .05]; Age [*F*_1.63, 45.50_ = 458.92, *p* < .001]; Interaction [*F*_1.63, 45.50_ = 0.63, *p* > .05].

Foxp2-MSNKO repeated measures ANOVA (untransformed): Genotype [*F*_1, 26_ = 0.24, *p* > .05]; Age [*F*_1.27, 32.99_ = 312.32, *p* < .001]; Interaction [*F*_1.27, 32.99_ = 0.12, *p* > .05].

Foxp2-CTXKO repeated measures ANOVA (untransformed): Genotype [*F*_1, 23_ = 0.03, *p* > .05]; Age [*F*_1.27, 29.09_ = 198.91, *p* < .001]; Interaction [*F*_1.27, 29.09_ = 0.12, *p* > .05].

**Adult weights**

2-tailed t-tests without corrections for multiple comparisons:

Foxp2-PCKO male: t_17_=0.33, *p* > .05.

Foxp2-PCKO female: t_17_=0.45, *p* > .05.

Foxp2-MSNKO male: t_22_=0.14 *p* > .05.

Foxp2-MSNKO female: t_15_=0.41 *p* > .05.

Foxp2-CTXKO male: t_15_=0.30 *p* > .05.

Foxp2-CTXKO female: t_18_=0.37 *p* > .05.

**Figure 2**

**(a) Reinforcer delivery rate**

Foxp2-PCKO FR8(1)-FR8(2s) repeated measures ANOVA (reciprocal data transformation): Genotype [*F*_1, 22_ = 4.48, *p* < .05]; Day [*F*_4.29, 94.48_ = 41.27, *p* < .001]; Interaction [*F*_4.29, 94.48_ = 3.41, *p* < .05].

Fisher’s LSD post hocs:

| 1 | 2 | 3 | 4 | 5 | 6 | 7 | 8 | 9 | 10 | 11 | 12 | 16s | 12s | 8s | 6s | 4s | 2s |
| --- | --- | --- | --- | --- | --- | --- | --- | --- | --- | --- | --- | --- | --- | --- | --- | --- | --- |
| ns | ns | ns | ns | ns | ns | ns | ns | ns | ns | ns | ns | *p* < .05 | *p* < .05 | *p* < .05 | *p* < .05 | *p* < .05 | ns |

Foxp2-PCKO FR8(16s)-FR8(2s) repeated measures ANOVA (reciprocal data transformation): Genotype [*F*_1, 22_ = 7.41, *p* < .05]; Day [*F*_3.01, 66.28_ = 43.82, *p* < .001]; Interaction [*F*_3.01, 66.28_ = 4.41,  *p* < .05].

Fisher’s LSD post hocs:

| 16s | 12s | 8s | 6s | 4s | 2s |
| --- | --- | --- | --- | --- | --- |
| *p* < .05 | ns | *p* < .05 | *p* < .05 | *p* < .05 | ns |

Foxp2-MSNKO FR8(1)-FR8(2s) repeated measures ANOVA (square root data transformation): Genotype [*F*_1, 19_ = 2.78, *p* > .05]; Day [*F*_4.30, 81.66_ = 16.11, *p* < .001]; Interaction [*F*_4.30, 81.66_ = 1.37, *p* > .05].

Foxp2-MSNKO FR8(16s)-FR8(2s) repeated measures ANOVA (square root data transformation): Genotype [*F*_1, 29_ = 6.20, *p* < .05]; Day [*F*_2.85, 54.06_ = 14.49, *p* < .001]; Interaction [*F*_2.85, 54.06_ = 0.18, *p* > .05].

Fisher’s LSD post hocs:

| 16s | 12s | 8s | 6s | 4s | 2s |
| --- | --- | --- | --- | --- | --- |
| ns | ns | ns | *p* < .05 | ns | ns |

Foxp2-CTXKO FR8(1)-FR8(2s) repeated measures ANOVA (reciprocal data transformation): Genotype [*F*_1, 32_ = 0.97, *p* > .05]; Day [*F*_5.10, 163.30_ = 25.93, *p* < .001]; Interaction [*F*_5.10, 163.30_ = 0.52, *p* > .05].

Foxp2-CTXKO FR8(16s)-FR8(2s) repeated measures ANOVA (reciprocal data transformation): Genotype [*F*_1, 32_ = 0.80, *p* > .05]; Day [*F*_2.85, 91.03_ = 22.09, *p* < .001]; Interaction [*F*_2.85, 91.03_ = 0.12, *p* > .05].

Controls FR8(1)-FR8(2s) repeated measures ANOVA (reciprocal data transformation): Control line [*F*_2, 37_ = 6.38, *p* < .05]; Day [*F*_5.49, 203.02_ = 55.13, *p* < .001]; Interaction [*F*_10.97, 203.02_ = 1.92, *p* < .05].

Fisher’s LSD post hocs:

|  | 1 | 2 | 3 | 4 | 5 | 6 | 7 | 8 | 9 | 10 | 11 | 12 | 16s | 12s | 8s | 6s | 4s | 2s |
| --- | --- | --- | --- | --- | --- | --- | --- | --- | --- | --- | --- | --- | --- | --- | --- | --- | --- | --- |
| Ctx vs Str | ns | ns | ns | ns | ns | *p* < .05 | *p* < .05 | ns | ns | *p* < .05 | *p* < .05 | ns | *p* < .05 | ns | ns | *p* < .05 | ns | ns |
| Ctx vs PC | ns | ns | ns | ns | *p* < .05 | *p* < .05 | *p* < .05 | *p* < .05 | *p* < .05 | *p* < .05 | *p* < .05 | *p* < .05 | *p* < .05 | *p* < .05 | *p* < .05 | *p* < .05 | ns | ns |
| Str vs PC | ns | ns | ns | ns | ns | ns | ns | ns | ns | ns | ns | *p* < .05 | *p* < .05 | *p* < .05 | ns | ns | ns | ns |

**Lever press rate**

Foxp2-PCKO FR8(1)-FR8(2s) repeated measures ANOVA (natural log data transformation): Genotype [*F*_1, 22_ = 4.00, *p* > .05]; Day [*F*_4.26, 93.76_ = 46.78, *p* < .001]; Interaction [*F*_4.26, 93.76_ = 1.49, *p* > .05].

Foxp2-PCKO FR8(16s)-FR8(2s) repeated measures ANOVA (natural log data transformation): Genotype [*F*_1, 22_ = 6.62, *p* < .05]; Day [*F*_2.52, 56.36_ = 1.12, *p* > .05]; Interaction [*F*_2.52, 56.36_ = 1.62,  *p* > .05].

Fisher’s LSD post hocs:

| 16s | 12s | 8s | 6s | 4s | 2s |
| --- | --- | --- | --- | --- | --- |
| *p* < .05 | ns | ns | *p* < .05 | *p* < .05 | ns |

Foxp2-MSNKO FR8(1)-FR8(2s) repeated measures ANOVA (natural log data transformation): Genotype [*F*_1, 19_ = 2.47, *p* > .05]; Day [*F*_4.31, 81.93_ = 25.75, *p* < .001]; Interaction [*F*_4.31, 81.93_ = 1.58, *p* > .05].

Foxp2-MSNKO FR8(16s)-FR8(2s) repeated measures ANOVA (natural log data transformation): Genotype [*F*_1, 19_ = 8.55, *p* < .05]; Day [*F*_2.00, 38.01_ = 2.20, *p* > .05]; Interaction [*F*_2.00, 38.01_ = 0.54,  *p* > .05].

Fisher’s LSD post hocs:

| 16s | 12s | 8s | 6s | 4s | 2s |
| --- | --- | --- | --- | --- | --- |
| ns | ns | ns | ns | *p* < .05 | *p* < .05 |

Foxp2-CTXKO FR8(1)-FR8(2s) repeated measures ANOVA (natural log data transformation): Genotype [*F*_1, 32_ = 1.14, *p* > .05]; Day [*F*_5.89, 188.50_ = 56.23, *p* < .001]; Interaction [*F*_5.89, 188.50_ = 0.72, *p* > .05].

Foxp2-CTXKO FR8(16s)-FR8(2s) repeated measures ANOVA (natural log data transformation): Genotype [*F*_1, 32_ = 0.85, *p* > .05]; Day [*F*_3.07, 98.26_ = 6.05, *p* = .001]; Interaction [*F*_3.07, 98.26_ = 0.56, *p* > .05].

Controls FR8(1)-FR8(2s) repeated measures ANOVA (natural log data transformation): Control line [*F*_2, 37_ = 6.33, *p* < .05]; Day [*F*_6.24, 231.02_ = 88.56, *p* < .001]; Interaction [*F*_12.49, 231.02_ = 1.96, *p* < .05].

Fisher’s LSD post hocs:

|  | 1 | 2 | 3 | 4 | 5 | 6 | 7 | 8 | 9 | 10 | 11 | 12 | 16s | 12s | 8s | 6s | 4s | 2s |
| --- | --- | --- | --- | --- | --- | --- | --- | --- | --- | --- | --- | --- | --- | --- | --- | --- | --- | --- |
| Ctx vs Str | ns | ns | ns | *p* < .05 | ns | *p* < .05 | *p* < .05 | ns | ns | *p* < .05 | *p* < .05 | ns | *p* < .05 | ns | ns | *p* < .05 | *p* < .05 | ns |
| Ctx vs PC | ns | *p* < .05 | ns | ns | *p* < .05 | *p* < .05 | *p* < .05 | *p* < .05 | *p* < .05 | *p* < .05 | *p* < .05 | *p* < .05 | *p* < .05 | *p* < .05 | *p* < .05 | *p* < .05 | *p* < .05 | ns |
| Str vs PC | ns | ns | ns | ns | ns | ns | ns | ns | ns | ns | ns | *p* < .05 | ns | *p* < .05 | ns | ns | ns | *p* < .05 |

**Efficiency**

Foxp2-PCKO repeated measures ANOVA (untransformed): Genotype [*F*_1, 22_ = 8.59, *p* < .05]; Day [*F*_2.83, 62.26_ = 246.59, *p* < .001]; Interaction [*F*_2.83, 62.26_ = 6.04, *p* = .001].

Fisher’s LSD post hocs:

| 16s | 12s | 8s | 6s | 4s | 2s |
| --- | --- | --- | --- | --- | --- |
| ns | *p* < .05 | *p* < .05 | *p* < .05 | *p* < .05 | ns |

Foxp2-MSNKO repeated measures ANOVA (untransformed): Genotype [*F*_1, 19_ = 2.41, *p* > .05]; Day [*F*_2.58, 48.97_ = 174.08, *p* < .001]; Interaction [*F*_2.58, 48.97_ = 0.42, *p* > .05].

Foxp2-CTXKO repeated measures ANOVA (untransformed): Genotype [*F*_1, 32_ = 1.23, *p* > .05]; Day [*F*_2.63, 84.01_ = 148.56, *p* < .001]; Interaction [*F*_2.63, 84.01_ = 0.35, *p* > .05].

**(c) Lever-press sequence analyses during self-paced training**

*Sequence length:*

Foxp2-PCKO repeated measures ANOVA (untransformed): Genotype [*F*_1, 22_ = 2.43, *p* > .05]; Day [*F*_4.73, 104.09_ = 27.78, *p* < .001]; Interaction [*F*_4.73, 104.09_ = 1.06, *p* > .05].

Foxp2-MSNKO repeated measures ANOVA (untransformed): Genotype [*F*_1, 19_ = 0.31, *p* > .05]; Day [*F*_11, 209_ = 34.92, *p* < .001]; Interaction [*F*_11, 209_ = 0.95, *p* > .05].

Foxp2-CTXKO repeated measures ANOVA (natural log data transformation): Genotype [*F*_1, 32_ = 2.18, *p* > .05]; Day [*F*_4.76, 152.34_ = 40.42, *p* < .001]; Interaction [*F*_4.76, 152.34_ = 0.54, *p* > .05].

*Sequence duration:*

Foxp2-PCKO repeated measures ANOVA (square root data transformation): Genotype [*F*_1, 22_ = 4.50, *p* < .05]; Day [*F*_11, 242_ = 21.53, *p* < .001]; Interaction [*F*_11, 242_= 1.19, *p* > .05].

Fisher’s LSD post hocs:

| 1 | 2 | 3 | 4 | 5 | 6 | 7 | 8 | 9 | 10 | 11 | 12 |
| --- | --- | --- | --- | --- | --- | --- | --- | --- | --- | --- | --- |
| ns | ns | *p* < .05 | ns | ns | ns | ns | ns | ns | *p* < .05 | ns | ns |

Foxp2-MSNKO repeated measures ANOVA (natural log data transformation): Genotype [*F*_1, 19_ = 5.04, *p* < .05]; Day [*F*_3.76, 71.39_ = 2.47, *p* > .05]; Interaction [*F*_3.76, 71.39_ = 0.90, *p* > .05].

Fisher’s LSD post hocs:

| 1 | 2 | 3 | 4 | 5 | 6 | 7 | 8 | 9 | 10 | 11 | 12 |
| --- | --- | --- | --- | --- | --- | --- | --- | --- | --- | --- | --- |
| ns | *p* < .05 | ns | ns | ns | ns | *p* < .05 | ns | ns | ns | ns | ns |

Foxp2-CTXKO repeated measures ANOVA (square root data transformation): Genotype [*F*_1, 32_ = 0.00, *p* > .05]; Day [*F*_6.31, 201.93_ = 14.15, *p* < .001]; Interaction [*F*_6.31, 201.93_ = 0.45, *p* > .05].

*Inter-sequence interval:*

Foxp2-PCKO repeated measures ANOVA (natural log data transformation): Genotype [*F*_1, 22_ = 1.14, *p* > .05]; Day [*F*_4.98, 109.44_ = 50.84, *p* < .001]; Interaction [*F*_4.98, 109.44_ = 3.01, *p* < .05].

Foxp2-MSNKO repeated measures ANOVA (natural log data transformation): Genotype [*F*_1, 19_ = 0.57, *p* > .05]; Day [*F*_5.22, 99.24_ = 12.19, *p* < .001]; Interaction [*F*_5.22, 99.24_ = 1.01, *p* > .05].

Foxp2-CTXKO repeated measures ANOVA (square root data transformation): Genotype [*F*_1, 32_ = 1.00, *p* > .05]; Day [*F*_6.65, 212.78_ = 15.28, *p* < .001]; Interaction [*F*_6.65, 212.78_ = 1.40, *p* > .05].

*Within-sequence IPI:*

Foxp2-PCKO repeated measures ANOVA (square root data transformation): Genotype [*F*_1, 22_ = 8.16, *p* < .01]; Day [*F*_4.34, 95.43_ = 76.83, *p* < .001]; Interaction [*F*_4.34, 95.43_ = 0.83, *p* > .05].

Fisher’s LSD post hocs:

| 1 | 2 | 3 | 4 | 5 | 6 | 7 | 8 | 9 | 10 | 11 | 12 |
| --- | --- | --- | --- | --- | --- | --- | --- | --- | --- | --- | --- |
| ns | *p* < .05 | *p* < .05 | *p* < .05 | *p* < .05 | *p* < .05 | ns | *p* < .05 | ns | *p* < .05 | ns | ns |

Foxp2-MSNKO repeated measures ANOVA (natural log data transformation): Genotype [*F*_1, 19_ = 4.45, *p* < .05]; Day [*F*_2.89, 54.84_ = 29.14, *p* < .001]; Interaction [*F*_2.89, 54.84_ = 0.36, *p* > .05].

Fisher’s LSD post hocs:

| 1 | 2 | 3 | 4 | 5 | 6 | 7 | 8 | 9 | 10 | 11 | 12 |
| --- | --- | --- | --- | --- | --- | --- | --- | --- | --- | --- | --- |
| ns | *p* < .05 | ns | ns | ns | ns | ns | ns | ns | ns | *p* < .05 | ns |

Foxp2-CTXKO repeated measures ANOVA (natural log data transformation): Genotype [*F*_1, 32_ = 0.37, *p* > .05]; Day [*F*_5.90, 188.80_ = 81.85, *p* < .001]; Interaction [*F*_5.90, 188.80_ = 0.48, *p* > .05].

**Figure 3**

**(b) Average median values**

*Rapid - ultrafast IPs:*

Foxp2-PCKO repeated measures ANOVA (reciprocal data transformation): Genotype [*F*_1, 22_ = 5.66, *p* < .05]; Day [*F*_4.19, 92.23_ = 33.08, *p* < .001]; Interaction [*F*_4.19, 92.23_ = 0.46, *p* > .05].

Fisher’s LSD post hocs:

| 1 | 2 | 3 | 4 | 5 | 6 | 7 | 8 | 9 | 10 | 11 | 12 | 16s | 12s | 8s | 6s | 4s | 2s |
| --- | --- | --- | --- | --- | --- | --- | --- | --- | --- | --- | --- | --- | --- | --- | --- | --- | --- |
| ns | ns | ns | ns | ns | ns | ns | *p* < .05 | ns | ns | ns | ns | ns | ns | *p* < .05 | *p* < .05 | ns | ns |

Foxp2-MSNKO repeated measures ANOVA (reciprocal data transformation): Genotype [*F*_1, 19_ = 2.91, *p* > .05]; Day [*F*_4.06, 77.16_ = 20.96, *p* < .001]; Interaction [*F*_4.06, 77.16_ = 0.52, *p* > .05].

Foxp2-CTXKO repeated measures ANOVA (reciprocal data transformation): Genotype [*F*_1, 32_ = 1.4, *p* > .05]; Day [*F*_6.39, 204.52_ = 38.77, *p* < .001]; Interaction [*F*_6.39, 204.52_ = 1.66, *p* > .05].

*Check IPIs:*

Foxp2-PCKO repeated measures ANOVA (natural log data transformation): Genotype [*F*_1, 22_ = 7.08, *p* < .05]; Day [*F*_2.18, 47.67_ = 35.94, *p* < .001]; Interaction [*F*_2.18, 47.67_ = 1.08, *p* > .05].

Fisher’s LSD post hocs:

| 1 | 2 | 3 | 4 | 5 | 6 | 7 | 8 | 9 | 10 | 11 | 12 | 16s | 12s | 8s | 6s | 4s | 2s |
| --- | --- | --- | --- | --- | --- | --- | --- | --- | --- | --- | --- | --- | --- | --- | --- | --- | --- |
| *p* < .05 | ns | ns | ns | *p* < .05 | *p* < .05 | *p* < .05 | *p* < .05 | ns | *p* < .05 | *p* < .05 | *p* < .05 | *p* < .05 | *p* < .05 | ns | *p* < .05 | *p* < .05 | ns |

Foxp2-MSNKO repeated measures ANOVA (reciprocal data transformation): Genotype [*F*_1, 19_ = 0.05, *p* > .05]; Day [*F*_4.41, 83.86_ = 37.46, *p* < .001]; Interaction [*F*_4.41, 83.86_ = 0.90, *p* > .05].

Foxp2-CTXKO repeated measures ANOVA (reciprocal data transformation): Genotype [*F*_1, 32_ = 5.78, *p* < .05]; Day [*F*_5.25, 168.13_ = 68.91, *p* < .001]; Interaction [*F*_5.25, 168.13_ = 1.16, *p* > .05].

Fisher’s LSD post hocs:

| 1 | 2 | 3 | 4 | 5 | 6 | 7 | 8 | 9 | 10 | 11 | 12 | 16s | 12s | 8s | 6s | 4s | 2s |
| --- | --- | --- | --- | --- | --- | --- | --- | --- | --- | --- | --- | --- | --- | --- | --- | --- | --- |
| ns | ns | ns | ns | ns | *p* < .05 | ns | *p* < .05 | ns | *p* < .05 | ns | *p* < .05 | ns | *p* < .05 | ns | *p* < .05 | ns | *p* < .05 |

*Consumption IPIs:*

Foxp2-PCKO repeated measures ANOVA (natural log data transformation): Genotype [*F*_1, 22_ = 8.09, *p* < .05]; Day [*F*_7.33, 161.32_ = 15.06, *p* < .001]; Interaction [*F*_7.33, 161.32_ = 1.10, *p* > .05].

Fisher’s LSD post hocs:

| 1 | 2 | 3 | 4 | 5 | 6 | 7 | 8 | 9 | 10 | 11 | 12 | 16s | 12s | 8s | 6s | 4s | 2s |
| --- | --- | --- | --- | --- | --- | --- | --- | --- | --- | --- | --- | --- | --- | --- | --- | --- | --- |
| ns | ns | ns | ns | ns | *p* < .05 | ns | *p* < .05 | *p* < .05 | ns | *p* < .05 | *p* < .05 | *p* < .05 | *p* < .05 | *p* < .05 | *p* < .05 | *p* < .05 | ns |

Foxp2-MSNKO repeated measures ANOVA (reciprocal square root data transformation): Genotype [*F*_1, 19_ = 0.70, *p* > .05]; Day [*F*_5.75, 109.31_ = 4.88, *p* < .001]; Interaction [*F*_5.75, 109.31_ = 0.63, *p* > .05].

Foxp2-CTXKO repeated measures ANOVA (reciprocal square root data transformation): Genotype [*F*_1, 32_ = 0.00, *p* > .05]; Day [*F*_6.97, 223.02_ = 6.76, *p* < .001]; Interaction [*F*_6.97, 223.02_ = 1.32, *p* > .05].

**(c) Average MAD / median values**

*Rapid - ultrafast IPIs:*

Foxp2-PCKO repeated measures ANOVA (natural log data transformation): Genotype [*F*_1, 22_ = 3.42, *p* > .05]; Day [*F*_6.70, 147.44_ = 8.69, *p* < .001]; Interaction [*F*_6.70, 147.44_ = 0.51, *p* > .05].

Foxp2-MSNKO repeated measures ANOVA (natural log data transformation): Genotype [*F*_1, 19_ = 16.47, *p* = .001]; Day [*F*_6.18, 117.47_ = 1.97, *p* > .05]; Interaction [*F*_6.18, 117.47_ = 0.40, *p* > .05].

Fisher’s LSD post hocs:

| 1 | 2 | 3 | 4 | 5 | 6 | 7 | 8 | 9 | 10 | 11 | 12 | 16s | 12s | 8s | 6s | 4s | 2s |
| --- | --- | --- | --- | --- | --- | --- | --- | --- | --- | --- | --- | --- | --- | --- | --- | --- | --- |
| *p* < .05 | ns | ns | ns | ns | ns | *p* < .05 | *p* < .05 | ns | ns | ns | *p* < .05 | ns | *p* < .05 | ns | ns | *p* < .05 | *p* < .05 |

Foxp2-CTXKO repeated measures ANOVA (natural log data transformation): Genotype [*F*_1, 32_ = 0.50, *p* > .05]; Day [*F*_7.78, 248.80_ = 7.34, *p* < .001]; Interaction [*F*_7.78, 248.80_ = 0.73, *p* > .05].

*Check IPIs:*

Foxp2-PCKO FR8(1)-FR8(2s) repeated measures ANOVA (reciprocal square root data transformation): Genotype [*F*_1, 22_ = 2.33, *p* > .05]; Day [*F*_5.96, 131.06_ = 8.20, *p* < .001]; Interaction [*F*_5.96, 131.06_ = 1.46, *p* > .05].

Foxp2-PCKO FR8(16s)-FR8(2s) repeated measures ANOVA (reciprocal square root data transformation): Genotype [*F*_1, 22_ = 2.63, *p* > .05]; Day [*F*_2.82, 62.00_ = 15.62, *p* < .001]; Interaction [*F* _2.82, 62.00_ = 1.18, *p* > .05].

Foxp2-MSNKO FR8(1)-FR8(2s) repeated measures ANOVA (natural log data transformation): Genotype [*F*_1, 19_ = 4.68, *p* < .05]; Day [*F*_6.36, 120.91_ = 5.93, *p* < .001]; Interaction [*F*_6.36, 120.91_ = 0.73, *p* > .05].

Fisher’s LSD post hocs:

| 1 | 2 | 3 | 4 | 5 | 6 | 7 | 8 | 9 | 10 | 11 | 12 | 16s | 12s | 8s | 6s | 4s | 2s |
| --- | --- | --- | --- | --- | --- | --- | --- | --- | --- | --- | --- | --- | --- | --- | --- | --- | --- |
| ns | ns | ns | ns | ns | ns | ns | ns | ns | ns | ns | ns | ns | ns | ns | ns | *p* < .05 | *p* < .05 |

Foxp2-MSNKO FR8(16s)-FR8(2s) repeated measures ANOVA (natural log data transformation): Genotype [*F*_1, 19_ = 7.68, *p* < .05]; Day [*F*_1.96, 37.31_ = 6.96, *p* < .01]; Interaction [*F*_1.96, 37.31_ = 0.32, *p* > .05].

Foxp2-CTXKO FR8(1)-FR8(2s) repeated measures ANOVA (reciprocal square root data transformation): Genotype [*F*_1, 32_ = 0.02, *p* > .05]; Day [*F*_9.34, 298.73_ = 9.76, *p* < .001]; Interaction [*F*_9.34, 298.73_ = 1.37, *p* > .05].

*Consumption IPIs:*

Foxp2-PCKO FR8(1)-FR8(2s) repeated measures ANOVA (untransformed): Genotype [*F*_1, 22_ = 3.43, *p* > .05]; Day [*F*_6.64, 146.02_ = 8.55, *p* < .001]; Interaction [*F*_6.64, 146.02_ = 1.09, *p* > .05].

Foxp2-PCKO FR8(16s)-FR8(2s) repeated measures ANOVA (untransformed): Genotype [*F*_1, 22_ = 5.72, *p* < .05]; Day [*F*_3.77, 83.89_ = 12.38, *p* < .001]; Interaction [*F*_3.77, 83.89_ = 1.48, *p* > .05].

Foxp2-MSNKO FR8(1)-FR8(2s) repeated measures ANOVA (untransformed): Genotype [*F*_1, 19_ = 1.26, *p* > .05]; Day [*F*_7.29, 138.43_ = 9.78, *p* < .001]; Interaction [*F*_7.29, 138.43_ = 0.52, *p* > .05].

Foxp2-MSNKO FR8(16s)-FR8(2s) repeated measures ANOVA (untransformed): Genotype [*F*_1, 19_ = 2.89, *p* > .05]; Day [*F*_5, 95_ = 10.17, *p* < .001]; Interaction [*F*_5, 95_ = 0.32, *p* > .05].

Foxp2-CTXKO FR8(1)-FR8(2s) repeated measures ANOVA (untransformed): Genotype [*F*_1, 32_ = 0.10, *p* > .05]; Day [*F*_7.85, 251.23_ = 7.50, *p* < .001]; Interaction [*F*_7.85, 251.23_ = 0.80, *p* > .05].

**Figure 4**

**ErasmusLadder**

*Foxp2-PCKO:*

Repeated measures ANOVA session 1-8: Genotype [*F*_1, 27_ = 9.25, *p* < .01]; Session [*F*_7, 116_ = 35.45, *p* < .001]; Interaction [*F*_7, 116_ = 1.58, *p* > .05].

Repeated measures ANOVA session 1-4: Genotype [*F*_1, 27_ = 8.15, *p* < .01]; Session [*F*_3, 81_ = 75.45, *p* < .001]; Interaction [*F*_3, 81_ = 2.89, *p* < .05].

Repeated measures ANOVA session 5-8: Genotype [F_1, 27_ = 5.96, *p* < .05]; Session [*F*_3, 81_ = 13.07, *p* < .001]; Interaction [*F*_3, 81_ = 0.51, *p* > .05].

*Foxp2-MSNKO:*

Repeated measures ANOVA session 1-8: Genotype [*F*_1, 14_ = 1.67, *p* > .05]; Session [*F*_7, 98_ = 9.68, *p* < .001]; Interaction [*F*_7, 98_ = 1.97, *p* > .05].

Repeated measures ANOVA session 1-4: Genotype [*F*_1, 14_ = 0.46, *p* > .05]; Session [*F*_3, 42_= 18.91, *p* < .001]; Interaction [*F*_3, 42_ = 1.20, *p* > .05].

Repeated measures ANOVA session 5-8: Genotype [*F*_1, 14_ = 5.35, *p* < .05]; Session [*F*_3, 42_ = 2.70, *p* > .05]; Interaction [*F*_3, 42_ = 0.40, *p* > .05].

*Foxp2-CTXKO:*

Repeated measures ANOVA session 1-8: Genotype [*F*_1, 27_ = 2.22, *p* > .05]; Session [*F*_7, 129_ = 25.66, *p* < .001]; Interaction [*F*_7, 129_ = 1.76, *p* > .05].

Repeated measures ANOVA session 1-4: Genotype [*F*_1, 27_ = 0.00, *p* > .05]; Session [*F*_3, 60_ = 50.36, *p* < .001]; Interaction [*F*_3, 60_ = 0.23, *p* > .05].

Repeated measures ANOVA session 5-8: Genotype [*F*_1, 27_ = 6.38, *p* < .05]; Session [*F*_3, 81_ = 13.69, *p* < .001]; Interaction [*F*_3, 81_ = 0.47, *p* > .05]

**Figure 5**

Foxp2-PCKO simple spike frequency at rest Mann-Whitney U test: *U* = 59.00, *p* < .05.

Foxp2-PCKO simple spike frequency during locomotion Mann-Whitney U test: *U* = 63.00, *p* < .05.

Foxp2-PCKO correlation between wheel velocity and simple spike activity Mann-Whitney U test: *U* = 162.00, *p* < .05.

Increased intrinsic excitability in PCs of Foxp2-PCKO mice. Repeated measures ANOVA Genotype [*F*_1, 20_ = 5.97, *p* < .05].
