# Supplementary material for: Differential effects of Foxp2 disruption in distinct motor circuits
Source: Mol Psychiatry. 2018 Aug 14;24(3):447–62. doi: 10.1038/s41380-018-0199-x (PMC6514880; doi:10.1038/s41380-018-0199-x)
Supplement: Supplementary file 2 — Supplementary Table 1 [file 41380_2018_199_MOESM2_ESM.docx]

**Supplementary Table 1.** Numbers of animals remaining in the high-speed training phase of the FR8 task.

| **Experimental Group** | **FR8 16 s** | **FR8 12s** | **FR8 8 s** | **FR8 6 s** | **FR8 4 s** | **FR8 2 s** |
| --- | --- | --- | --- | --- | --- | --- |
| Foxp2-PCKO Ctr | 100% (13/13) | 100% (13/13) | 100% (13/13) | 100% (13/13) | 100% (13/13) | 100% (13/13) |
| Foxp2-PCKO | 100% (11/11) | 100% (11/11) | 100% (11/11) | 100% (11/11) | 100% (11/11) | 81.8% (9/11) |
| Foxp2-MSNKO Ctr | 100% (11/11) | 100% (11/11) | 100% (11/11) | 100% (11/11) | 100% (11/11) | 100% (11/11) |
| Foxp2-MSNKO | 100% (10/10) | 100% (10/10) | 100% (10/10) | 100% (10/10) | 100% (10/10) | 100% (10/10) |
| Foxp2-CTXKO Ctr | 100% (16/16) | 100% (16/16) | 100% (16/16) | 100% (16/16) | 100% (16/16) | 87.5% (14/16) |
| Foxp2-CTXKO | 100% (18/18) | 100% (18/18) | 100% (18/18) | 94.4% (17/18) | 94.4% (17/18) | 77.7% (14/18) |
