# Supplementary material for: Differential effects of Foxp2 disruption in distinct motor circuits
Source: Mol Psychiatry. 2018 Aug 14;24(3):447–62. doi: 10.1038/s41380-018-0199-x (PMC6514880; doi:10.1038/s41380-018-0199-x)
Supplement: Supplementary file 3 — Supplementary Table 2 [file 41380_2018_199_MOESM3_ESM.docx]

**Supplementary Table 2.** Summary of cell count data.

| **Experimental Group** | ***n*** | **Purkinje cells/ 0.5 mm (average of lobules III, VI and IX)** | | |  | **Striatal cells (sum of 2 fields)** | | |  | **Cortical cells/ (sum of 2 fields)** | | |
| --- | --- | --- | --- | --- | --- | --- | --- | --- | --- | --- | --- | --- |
|  |  | **Mean** | **SD** | **t-test** |  | **Mean** | **SD** | **t-test** |  | **Mean** | **SD** | **t-test** |
| Foxp2-PCKO Ctr | 4 | 12 | 3 | *t*_6_ = 2.04, *p* > .05 |  | 107 | 14 | *t*_5_ = 0.54, *p* > .05 |  | 104 | 21 | *t*_5_ = 1.08, *p* > .05 |
| Foxp2-PCKO | 5 | 7 | 3 |  |  | 119 | 43 |  |  | 119 | 15 |  |
| Foxp2-MSNKO Ctr | 4 | 12 | 4 | *t*_6_ = 0.43, *p* > .05 |  | 149 | 26 | *t*_6_ = 2.02, *p* > .05 |  | 125 | 18 | *t*_6_ = 1.50, *p* > .05 |
| Foxp2-MSNKO | 4 | 14 | 4 |  |  | 117 | 8 |  |  | 151 | 25 |  |
| Foxp2-CTXKO Ctr | 3 | 12 | 3 | *t_4_* = 0.48, *p* > .05 |  | 110 | 14 | *t_4_* = 0.40, *p* > .05 |  | 112 | 3 | *t_4_* = 0.98, *p* > .05 |
| Foxp2-CTXKO | 3 | 14 | 6 |  |  | 104 | 18 |  |  | 100 | 18 |  |
