# Supplementary figures and images for: Differential effects of Foxp2 disruption in distinct motor circuits
Source: Mol Psychiatry. 2018 Aug 14;24(3):447–62. doi: 10.1038/s41380-018-0199-x (PMC6514880; doi:10.1038/s41380-018-0199-x)

Control 1

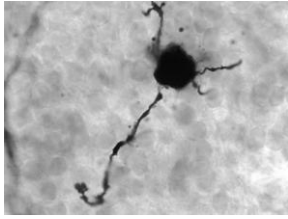

Control 2

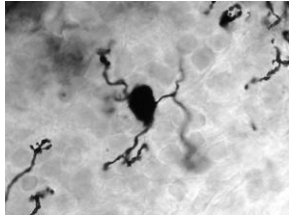

Foxp2-PCKO 1

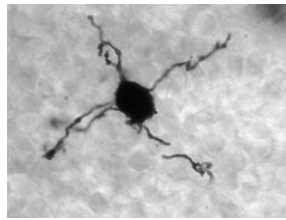

Foxp2-PCKO 2

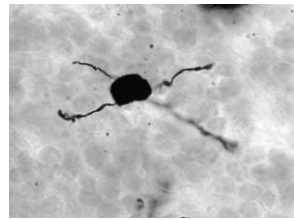

Control

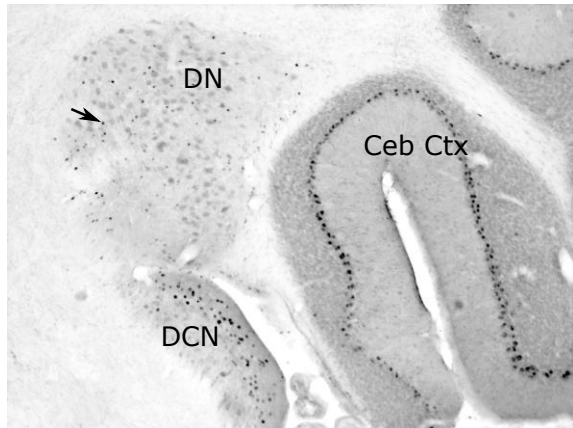

Foxp2-PCKO

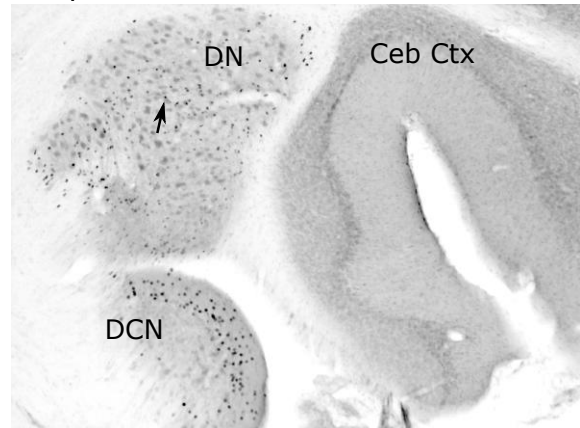

Supplement: Supplementary file 4 — Supplementary Figure 1 [file 41380_2018_199_MOESM4_ESM.pdf]

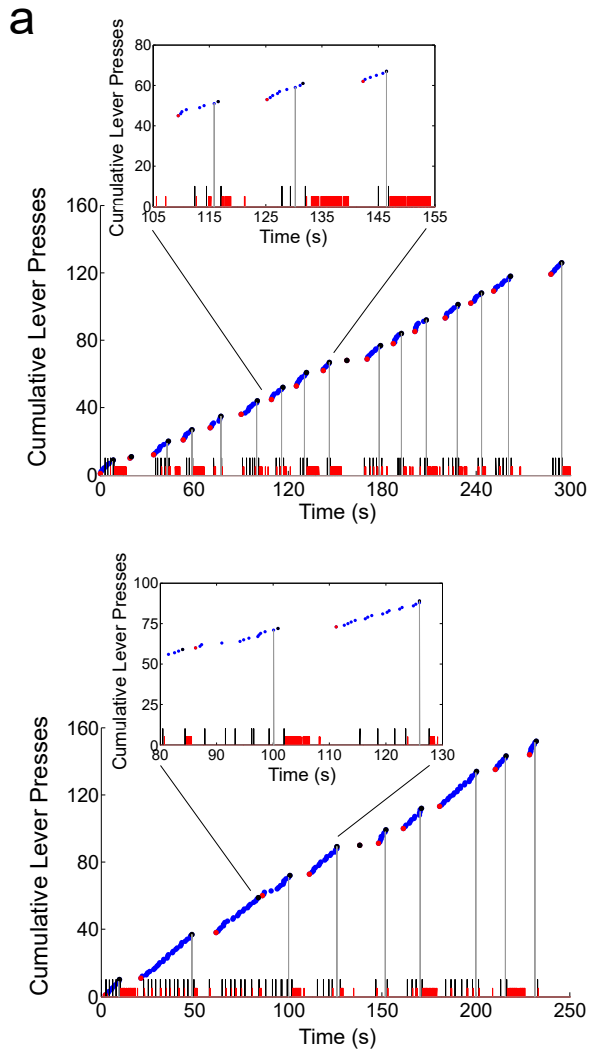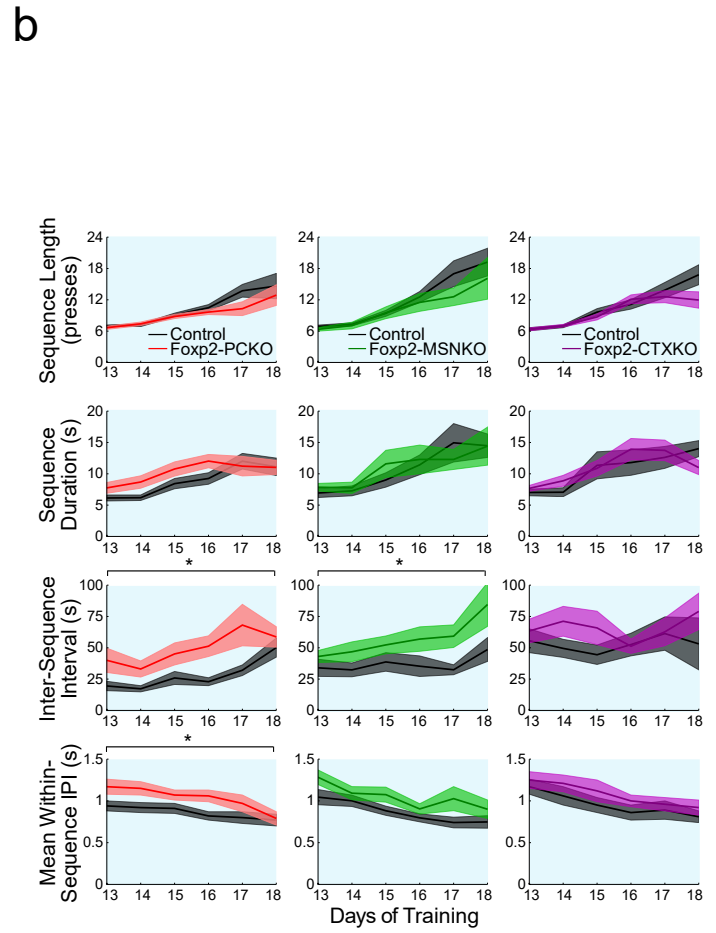

Supplement: Supplementary file 5 — Supplementary Figure 2 [file 41380_2018_199_MOESM5_ESM.pdf]

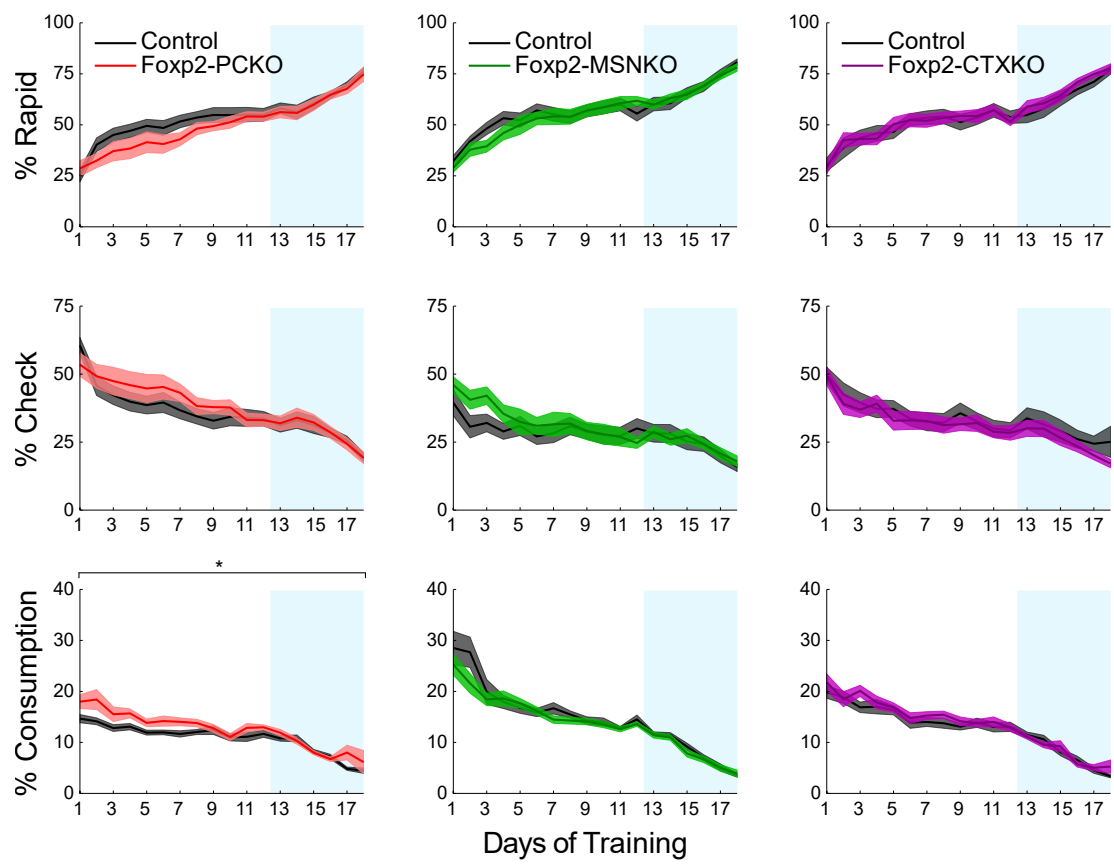

Supplementary Figure 3

Supplement: Supplementary file 6 — Supplementary Figure 3 [file 41380_2018_199_MOESM6_ESM.pdf]

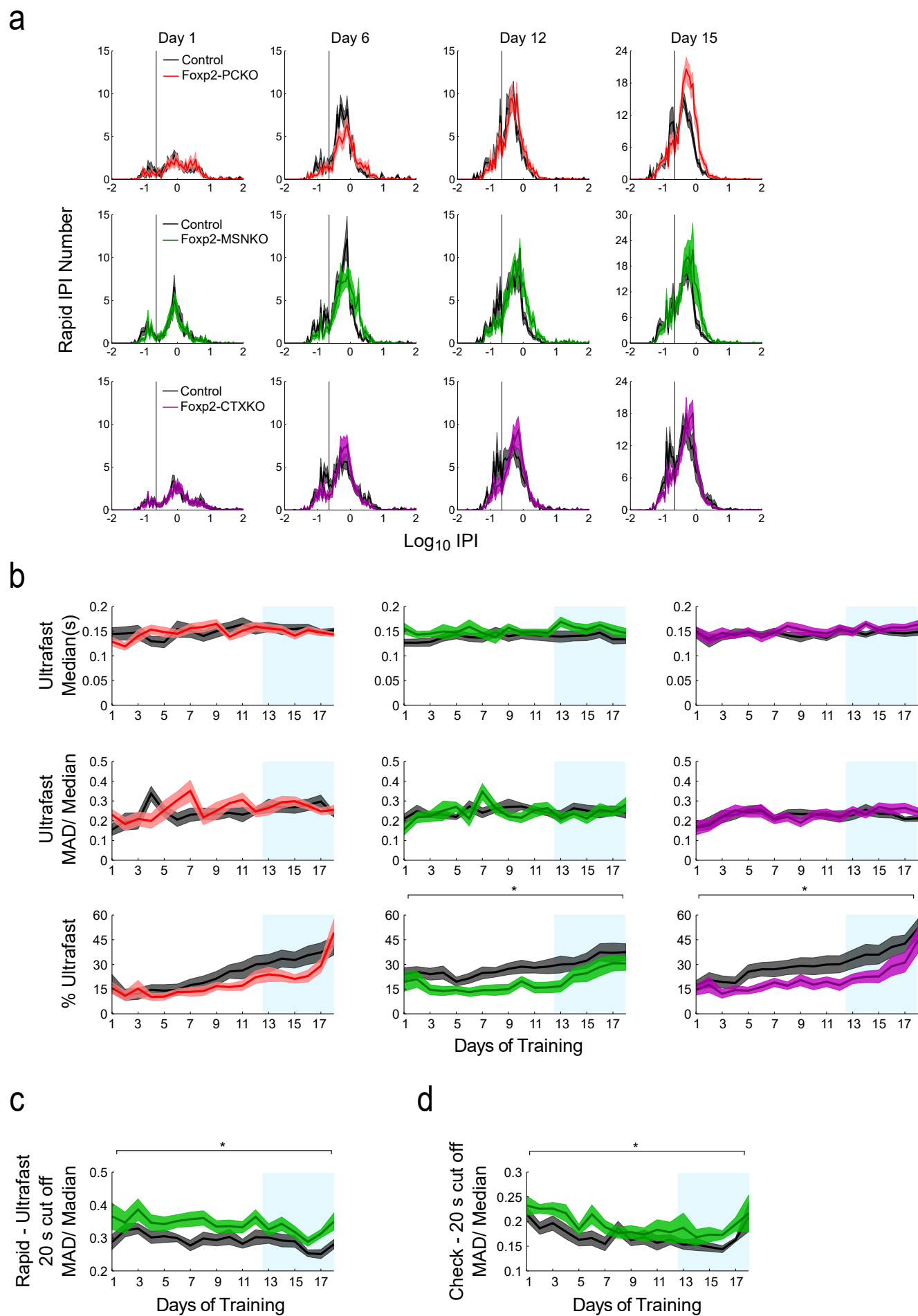

Supplementary Figure 4

Supplement: Supplementary file 7 — Supplementary Figure 4 [file 41380_2018_199_MOESM7_ESM.pdf]

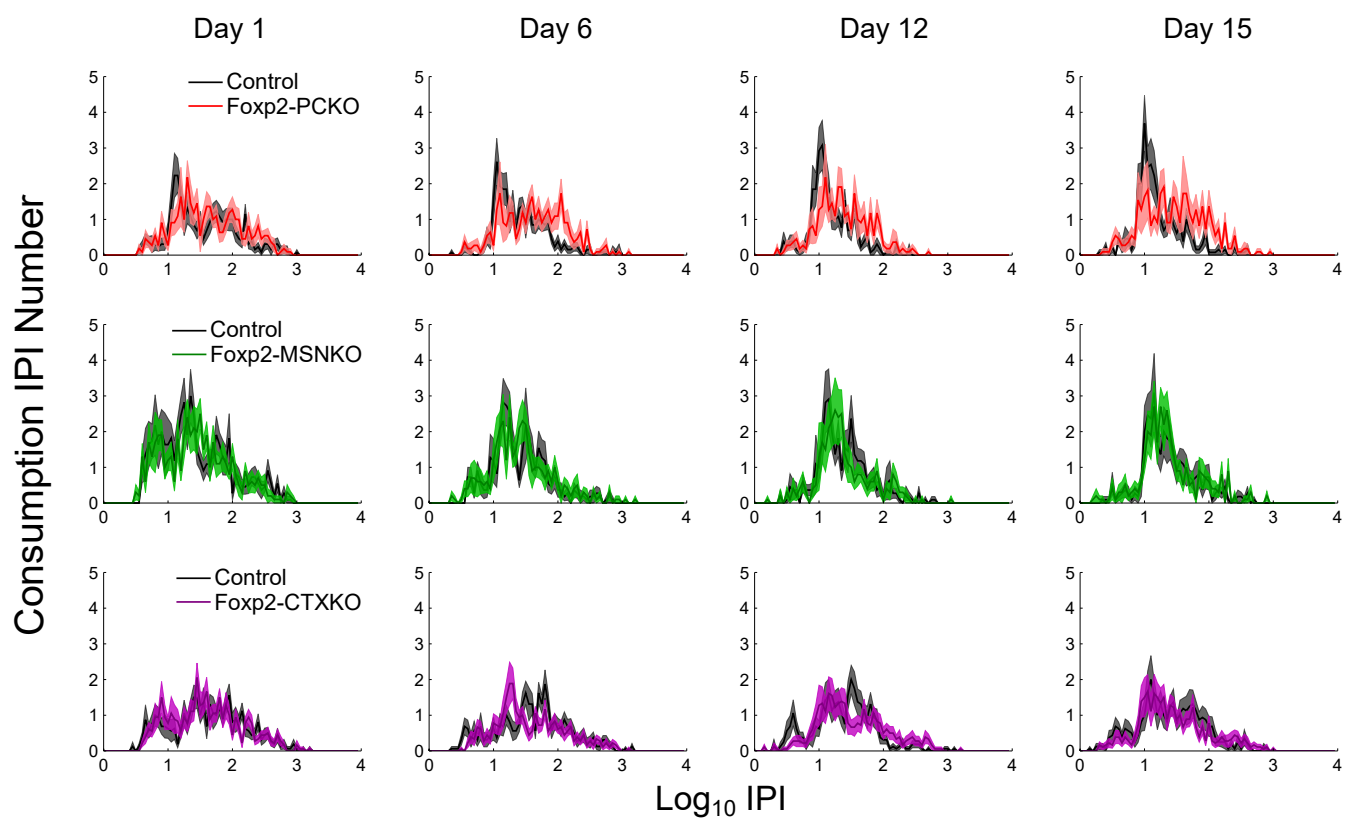

Supplementary Figure 5

Supplement: Supplementary file 8 — Supplementary Figure 5 [file 41380_2018_199_MOESM8_ESM.pdf]

a

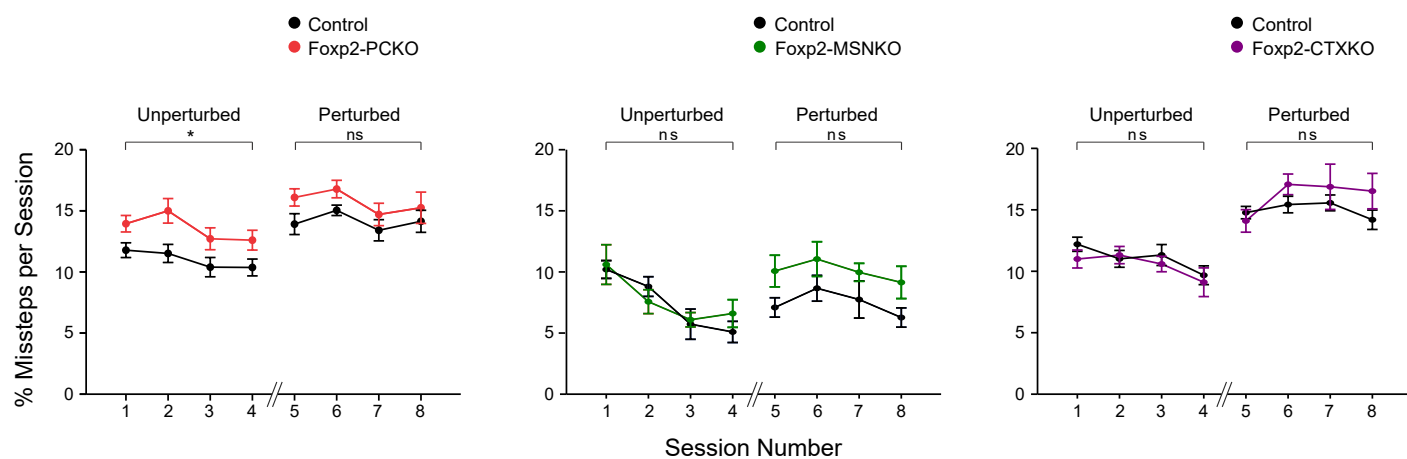

b

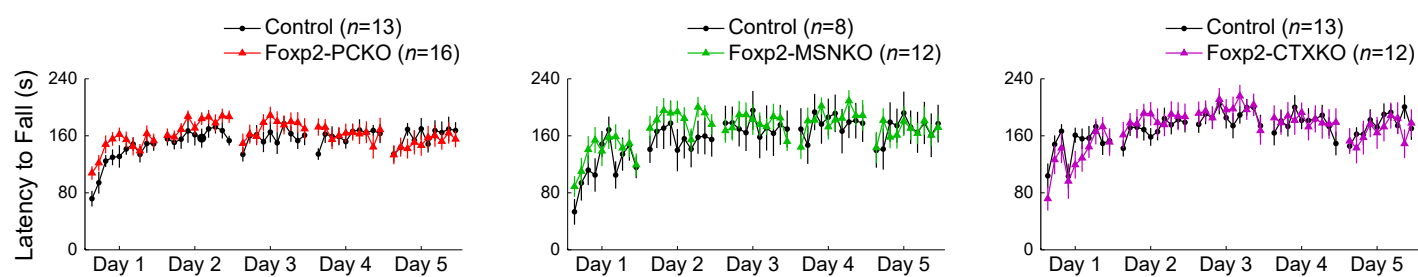

Supplement: Supplementary file 9 — Supplementary Figure 6 [file 41380_2018_199_MOESM9_ESM.pdf]

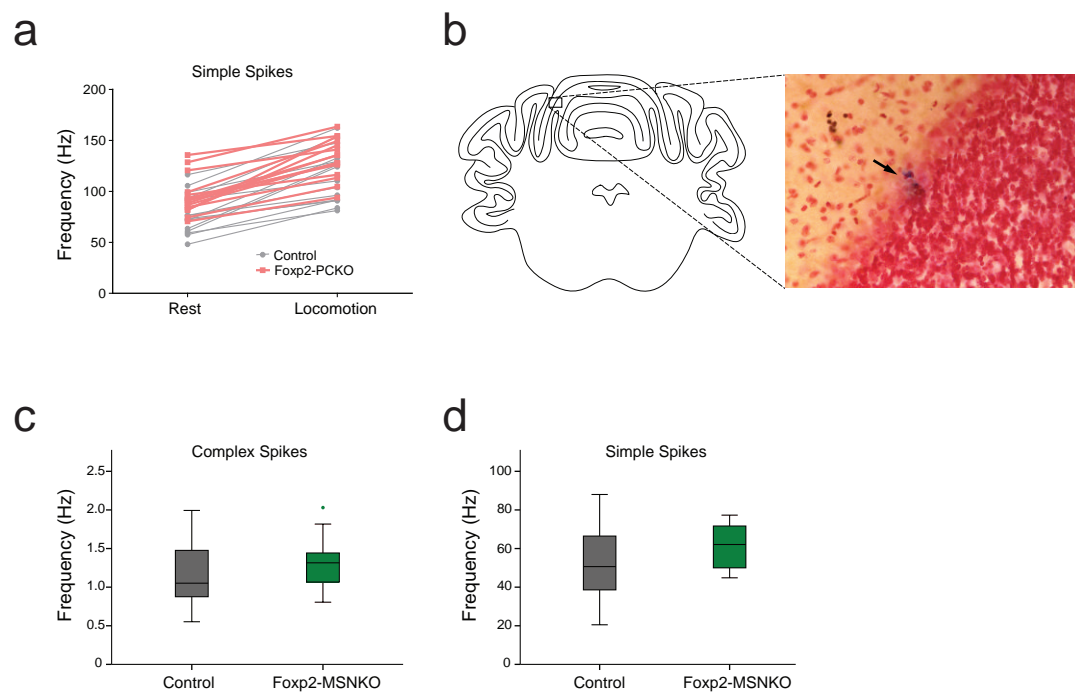

Supplement: Supplementary file 10 — Supplementary Figure 7 [file 41380_2018_199_MOESM10_ESM.pdf]

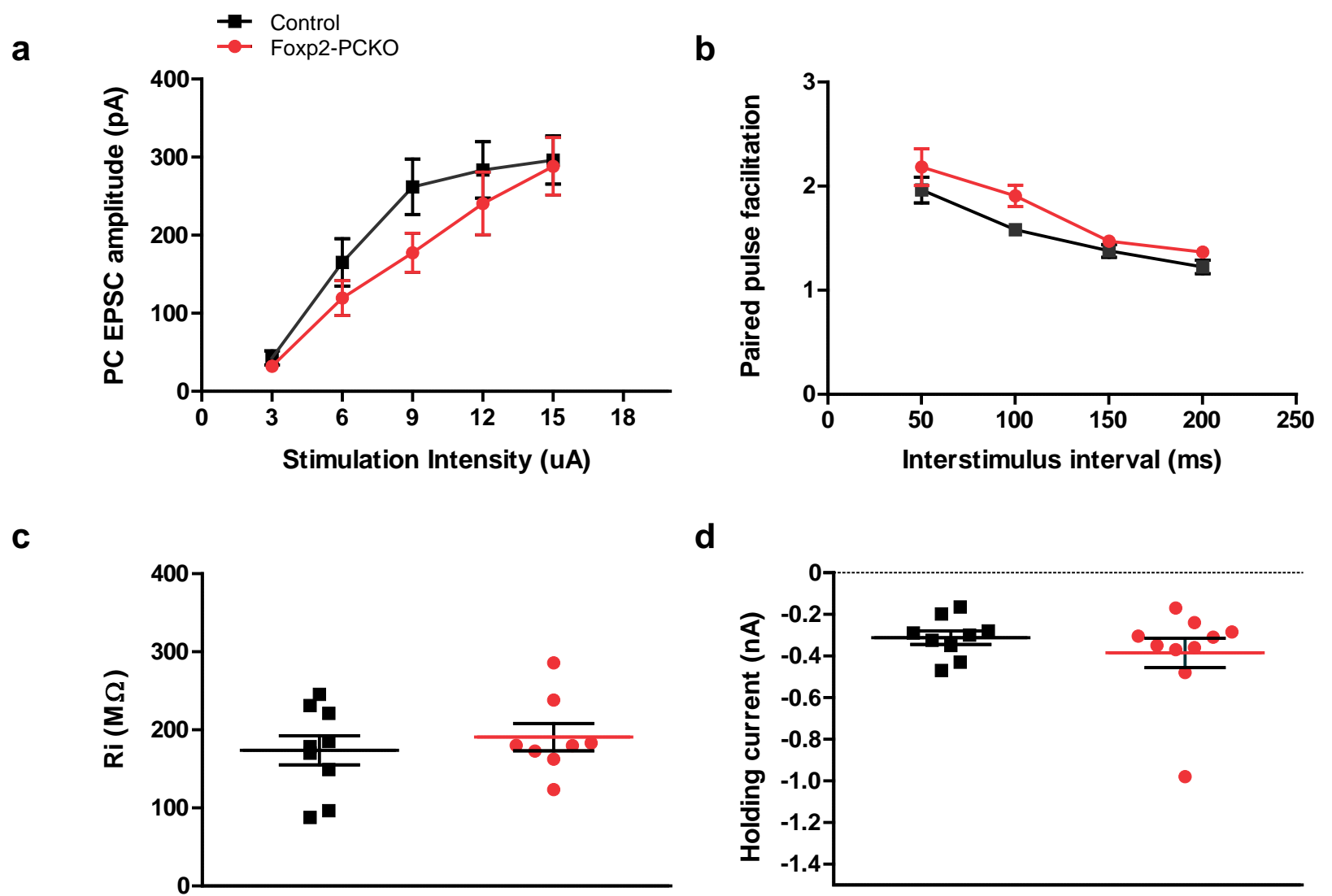

Supplement: Supplementary file 11 — Supplementary Figure 8 [file 41380_2018_199_MOESM11_ESM.pdf]

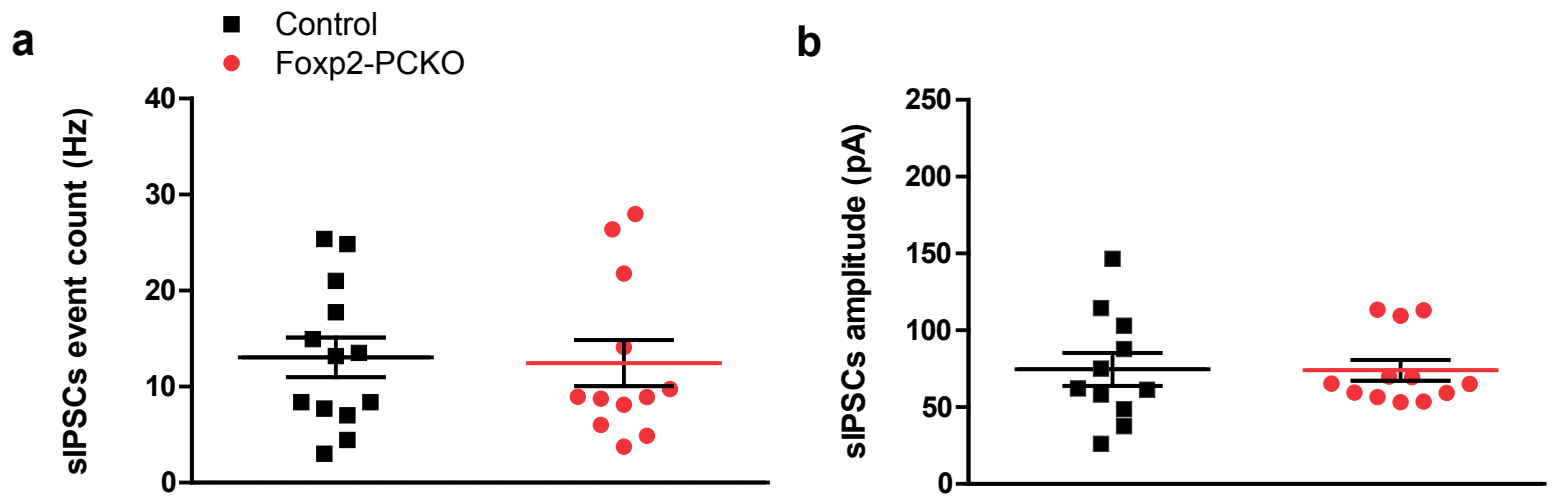

Supplement: Supplementary file 12 — Supplementary Figure 9 [file 41380_2018_199_MOESM12_ESM.pdf]

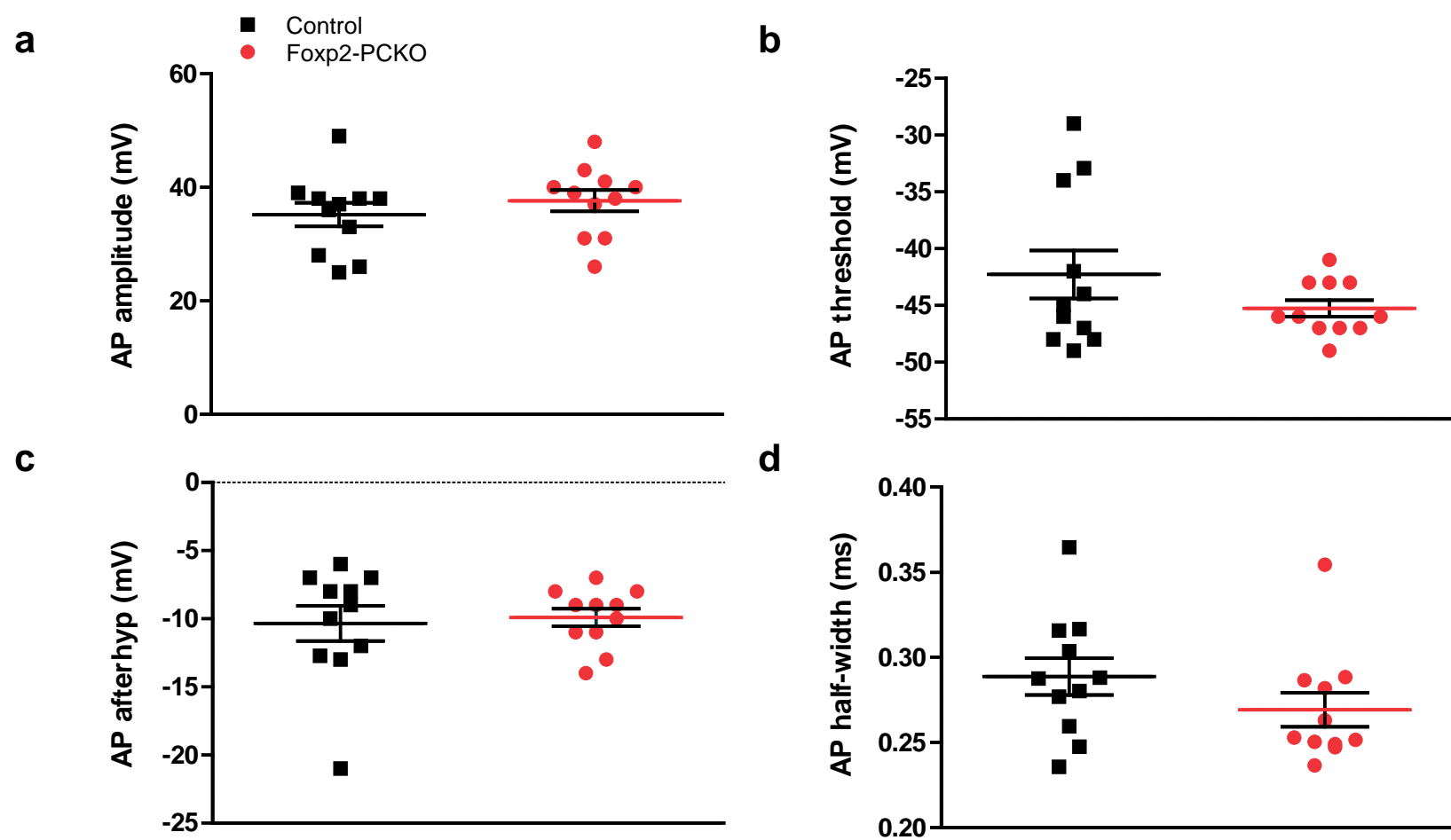

Supplement: Supplementary file 13 — Supplementary Figure 10 [file 41380_2018_199_MOESM13_ESM.pdf]
